# Growth Phase-Related DNA Methylation and Localized DNA Fragment Density Variation in *Escherichia coli* and *Lactobacillus acidophilus* Genomes

**DOI:** 10.3390/epigenomes10030057

**Published:** 2026-09-11

**Authors:** Ziming Chen, Chian Teng Ong, David Chau, Elizabeth M. Ross

**Affiliations:** Queensland Alliance for Agriculture and Food Innovation, The University of Queensland, St. Lucia, QLD 4072, Australia; chianteng.ong@uq.edu.au (C.T.O.); suiki.chau@uq.edu.au (D.C.); e.ross@uq.edu.au (E.M.R.)

**Keywords:** bacteria, growth phase, DNA methylation, long-read sequencing

## Abstract

**Background/Objectives:** Bacterial DNA methylation is a reversible genetic modification affecting gene regulation without significant DNA sequence alteration, thereby enabling rapid adaptation to various environments. However, traditional motif-based approaches detect DNA methylation at low resolution, limiting the identification of DNA methylation variation and often restricting analysis of multiple methylation forms. **Methods:** Here, we used Oxford Nanopore Technologies sequencing to examine genome-wide variation in three DNA methylation forms (N6-methyladenine, N4-methylcytosine, and 5-methylcytosine) and DNA fragment density between the exponential and stationary phases of *Escherichia coli* and *Lactobacillus acidophilus*. **Results:** During the exponential phase, regions surrounding the replication terminus displayed lower DNA fragment density in both species and lower 5-methylcytosine levels in *Escherichia coli*. Methylation calling on each genomic locus revealed almost 21% more differentially methylated sites than motif-based detection. Through the locus-based detection, the majority (72%) of differentially methylated genes in *Escherichia coli* were differentially expressed. The functions of these genes were associated with the physiological response to growth phase transition, such as cell membrane synthesis and translation. Incorporating multiple DNA methylation forms and including all methylated loci, not just those within common motifs, resulted in higher concordance between differentially expressed genes and differentially methylated genes compared to single-form or motif-based approaches. **Conclusions:** Our findings suggest that the bacterial DNA methylation pattern is affected by growth phase transition, and that a locus-based differential methylation analysis using Nanopore sequencing can estimate microbial activity in some cases, especially in microbiome studies where the transcriptome-genome link is difficult to establish. **Impact Statement:** Bacterial DNA methylation is a reversible genetic modification affecting gene regulation, enabling rapid adaptation. There are three major DNA methylation forms in bacteria, namely N6-methyladenine, N4-methylcytosine, and 5-methylcytosine. Using Oxford Nanopore sequencing, we found that DNA methylation levels (N6-methyladenine and 5-methylcytosine) are globally and regionally lower during the exponential phase compared to the stationary phase. Our results showed that differentially methylated genes can robustly estimate the overall transcriptomic profile in *Escherichia coli*. The inclusion of multiple DNA methylation forms and all methylated loci can improve concordance with differentially expressed genes. Our findings reveal that the bacterial DNA methylation pattern shifts during growth phase transition, and indicate that methylation could be an accessible method for estimating microbial activity.

## 1. Introduction

Bacterial DNA methylation is an epigenetic tool for genome defense against foreign DNA invasion and gene regulation [1,2,3]. Unlike mutations permanently altering genetic sequences, DNA methylation regulates gene expression with reversible genetic changes, thereby preventing the deleterious effects on cell line propagation. N6-methyladenine (m6A), N4-methylcytosine (m4C), and 5-methylcytosine (m5C) are three common DNA methylation forms in bacteria, with m6A as the most abundant, followed by m4C [4]. During the methylation process, DNA methylases catalyze the addition of the methyl group to the DNA bases. In eukaryotes, the added methyl group of the nucleotide can recruit gene repressors or disrupt the binding between DNA and transcription factors [5], eventually affecting gene expression and phenotypes. In bacteria, the methylation patterns in regulatory regions, such as operons and promoters, also influence gene expression [6,7].

The introduction of microbial cultivation in the mid-1800s has extensively advanced the study of microbial growth and physiology [8]. The classic growth cycle of bacteria during the batch culture consists of four phases: lag, exponential (or log), stationary, and death [8]. Among these, the exponential and stationary phases are the most comprehensively studied and characterized. Generally, in the exponential phase, the population of bacterial cells grows exponentially in nutrient-rich environments; in contrast, the stationary phase is marked by a stable cell number because of nutrient availability stress. The transition into the stationary phase requires bacterial adaptations in their metabolism and morphology, regulated by the sigma factor and multiple regulators [9]. However, the variation in bacterial DNA methylation during growth phase transition or under nutritional limitation largely remains unexplored, particularly for multiple methylation types at single-nucleotide resolution. This gap likely arises because tools for genome-wide methylation detection have been historically targeted towards eukaryotic methylation, especially m5C, mostly ignoring other methylation forms.

Previous DNA methylation detection approaches, such as bisulfite sequencing and Sanger sequencing, require enzymatic or chemical conversion and can only identify one DNA modification form in each run [4], which limits the comprehensive bacterial DNA methylation analysis. Currently, single-molecule real-time sequencing (SMRT) and Oxford Nanopore Technologies (ONT) sequencing, the third-generation long-read sequencing techniques, can simultaneously detect multiple DNA modification forms without the necessity of enzymatic or chemical conversion [4]. During sequencing, ONT can distinguish the deviated signals of the methylated DNA base from the canonical equivalent, which allows the identification of DNA methylation at the single-nucleotide level [10]. The availability of third-generation sequencing provides new opportunities to research prokaryotic DNA methylation and prokaryotic meta-epigenomics, e.g., [11,12].

Studies to date have used third-generation sequencing to characterize bacterial DNA methylation patterns under different conditions using motif identification and genome-level motif frequencies [13,14,15,16]. However, these motif-related approaches may fail to reveal the DNA methylome difference in the absence of genetic variants, and they are limited to different background factors, such as sequencing depth and GC content. These motif-based methods can also overlook rare DNA methylation motifs that are likely undetectable due to the low coverage or methylation rates, leading to methylome difference misestimation. The methylation detection through third-generation sequencing can overcome these limitations. They provide a more comprehensive DNA methylation analysis targeting each genomic locus (motif-free), allowing the capture of most bacterial DNA methylation variance.

This study aimed to characterize localized DNA fragment density and DNA methylation variation (m6A, m4C, and m5C) across genomes in bacteria (*Escherichia coli* and *Lactobacillus acidophilus*) between two of the most dramatically different conditions of bacterial growth: exponential and stationary phases, using Oxford Nanopore sequencing. Transcriptomic profiles were also incorporated to examine the association between bacterial DNA methylation and gene expression.

## 2. Results

In this study, two bacterial species, *Escherichia coli* and *Lactobacillus acidophilus*, were utilized to examine the DNA read length, localized DNA fragment density, and DNA methylation variation between the exponential and stationary phases using Oxford Nanopore sequencing, with gene expression profiles additionally evaluated in *E. coli*. Consistent with the growth curves that we calculated (Figures S1 and S2), *E. coli* is generally considered fast-growing, while *L. acidophilus* is slow-growing; this contrast allowed us to examine the localized DNA fragment density and methylation patterns in bacteria with different growth rates. Bacterial suspensions from the exponential and stationary phases (Table 1) for paired DNA and RNA extraction were collected from the same biological replicate to reduce biological variability. Our study first determined the required sequencing depth, followed by analyses of DNA fragment density, motif identification, methylation comparisons, and transcriptomic integration.

### 2.1. Sequencing Depth Determination for DNA Methylation Characterization

The genome assembly was first performed using samples from the stationary phase. The completeness of the assembled *E. coli* and *L. acidophilus* genomes was 96.55% and 93.97%, respectively (Table S7). The assembled genomes of *E. coli* and *L. acidophilus* shared 97.63% and 99.99% similarity with their corresponding NCBI RefSeq genomes (*E. coli*: GCF_000008865; *L. acidophilus*: GCF_003047065.1), respectively. The assembled and NCBI RefSeq genomes were used for DNA methylation calling and sequencing depth determination.

**Figure 1:**
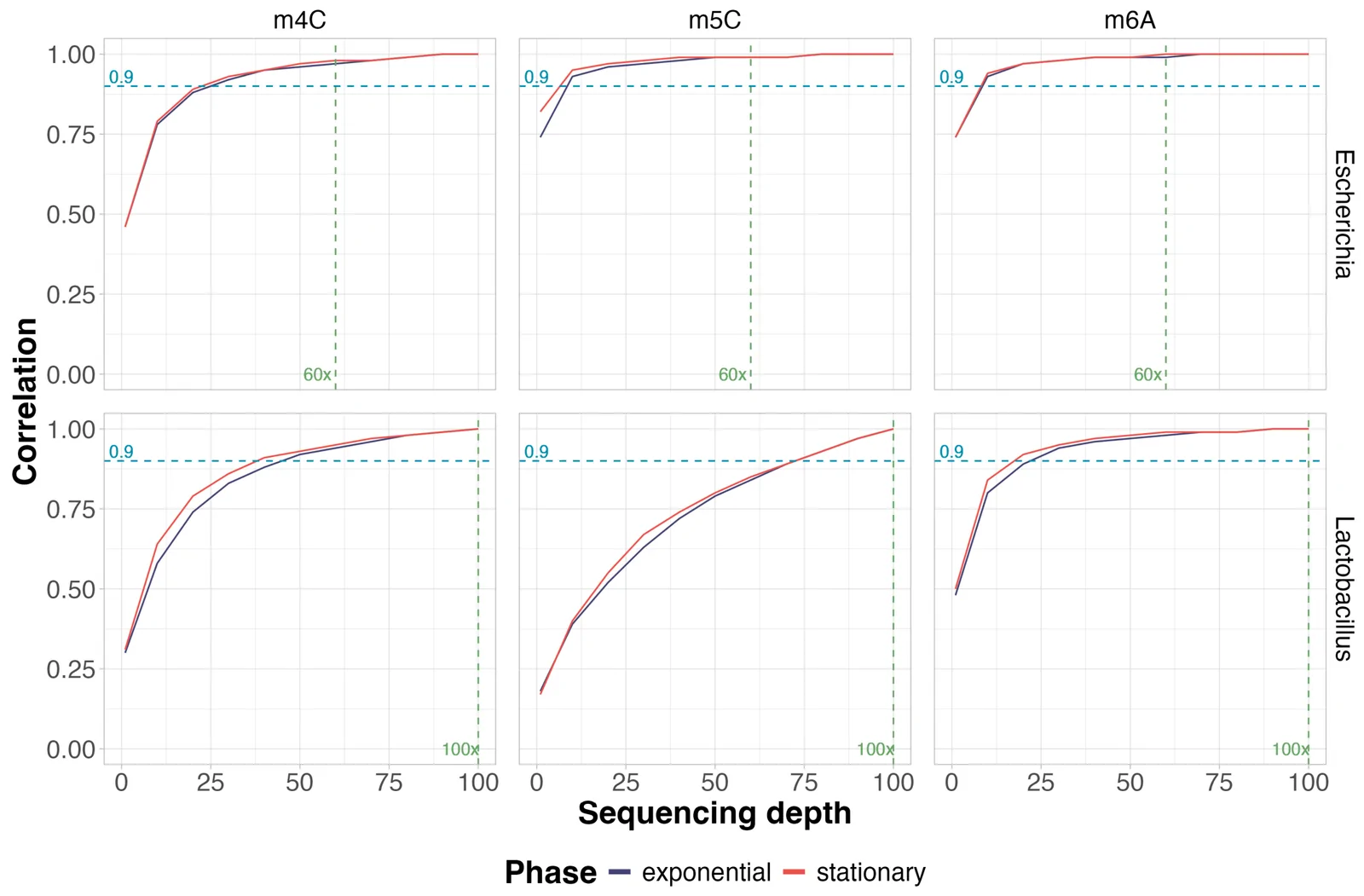
Base-level DNA methylation correlations of different sequencing depths with 100× coverage across three DNA methylation. The RefSeq genomes were used for the analysis. Only samples with over 100× coverage in the dataset were used for this analysis. Samples with 100× coverage were used as a benchmark. Genomic loci present in all subsampled datasets were used without specifying motifs. The Pearson correlation coefficient was calculated to evaluate the correlation between different sequencing depths with 100× coverage using the base-level DNA methylation proportions. m6A: N6-methyladenine. m4C: N4-methylcytosine. m5C: 5-methylcytosine.

Each adenine or cytosine base on both strands in the genome (locus-based) was included in this analysis to inform three methylation types (m6A, m4C, and m5C). The minimum sequencing depth for characterizing each type of DNA methylation was determined using the subsampled data (1×, 10×, 20×, …, 100×). Samples with above 100× coverage were used in this analysis (*E. coli*: three of five biological samples; *L. acidophilus*: three biological samples). The 100× coverage was used as a benchmark for DNA methylation characterization. In the *E. coli* dataset, 60× coverage demonstrated a strong correlation to 100× coverage (R > 0.9) across all modification types (Figure 1 and Figure S19). In comparison, low correlations (R < 0.9) were observed in the m5C of the *L. acidophilus* dataset when the sequencing coverage was lower than 70× (RefSeq genome) and 60× (assembled genome). This indicated that 60× coverage was sufficient to reveal most methylation variation in *E. coli*, while a coverage over 70× was adequate for characterizing the m6A and m4C pattern in *L. acidophilus*. Therefore, to balance computational efficiency and profile stability, in the subsequent analyses, the corresponding sequencing coverage for each *E. coli* or *L. acidophilus* dataset was 60× and 100×, respectively. However, we noted that the m5C profiling in *L. acidophilus* likely requires sequencing depths over 100× for DNA methylation detection, potentially because the available DNA methylation ONT training models do not include *L. acidophilus* [17] or the low occurrence of m5C methylation in *L. acidophilus*.

### 2.2. Shorter DNA During the Exponential Phase

**Figure 2:**
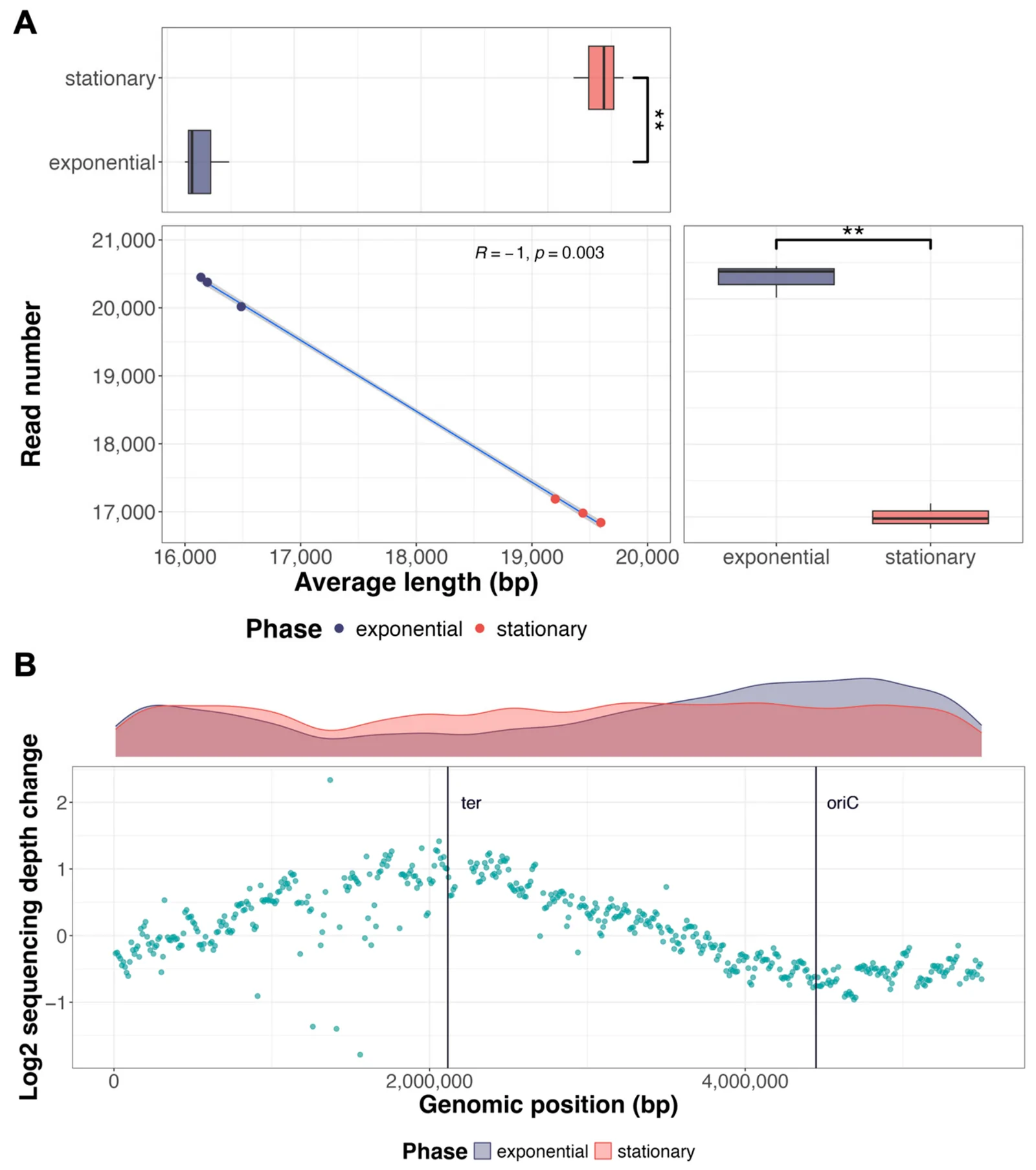
DNA fragment density analyses of *Escherichia coli*. (**A**) Average DNA length and read number between the exponential and stationary phases. (**B**) Sequencing depth changes in each 10 kb window across the genome between the exponential and stationary phases. Analysis here only included replicates 1, 2, and 3 with DNA and RNA sequencing data in *E. coli*. The side plot on the top of graph (**B**) is the DNA sequence start site density across the reference genome. The RefSeq genome (GCF_000008865) was used for the analysis. Only the data from the chromosome NC_002695.2 were included in graph (**B**). The log2 value change is the base-2 logarithm of the stationary value relative to the exponential value. The Spearman correlation coefficient was calculated to estimate the correlation between average DNA length and read number. A paired *t*-test was used to compare the average length and read number between different phases. *ter*: DNA replication terminus. *oriC*: DNA replication origin. *p*-values < 0.01: **.

Multiple DNA strands are undergoing replication in the exponential phase because of active cell division and DNA synthesis. Therefore, we hypothesized that we would observe a higher DNA fragment number and shorter fragments in the exponential phase compared to the stationary phase. The primary mapped reads were used for the analysis. Consistent with our hypothesis, significantly shorter average read lengths were observed in the exponential phase of *E. coli*, compared with the stationary phase (*p* < 0.01; Figure 2A). Likewise, the shorter reads were recorded in the exponential phase of *L. acidophilus*, compared to the stationary phase (*p* < 0.05; Figure S8A).

### 2.3. Localized DNA Fragment Density Variation During the Exponential Phase

The active cell division during the exponential phase could also result in frequent DNA replication initiation. We hypothesized that DNA molecules were enriched around the DNA replication initiation origin, contributing to the uneven sequencing depth distribution across the genome following DNA sequencing. We used the sequencing depth as a proxy for the relative number of DNA fragments present, where each sequencing read represented an independent piece of DNA in the sample. Nanopore sequencing only sequences each DNA molecule once when using the amplification-free protocols, such as the one used here. This enables us to calculate the relative proportions of DNA from different genome regions by comparing the sequencing depth. Previous work [12] showed that there was no systematic bias between the sequencing depth across genome locations when using ligation-based Nanopore protocols, suggesting that the depth reliably reflects the genome-wide DNA fragment density present.

The sequencing depth of each 10 kb genome window and the distribution of read start sites (read end sites for reverse strand) across the genome between the exponential and stationary phases were compared. Each program was run twice, once with the NCBI RefSeq genome, and once with the assembled genome as a reference. The DNA fragment density across different locations of the genome was determined by the sequencing bases at each respective location, assuming no localized bias during sequencing. The read start site was considered the DNA replication start site for the newly synthesized leading/lagging DNA strands. In *E. coli*, higher sequencing depths were observed in regions surrounding the DNA replication origin (*oriC*) in the exponential phase (Figure 2B and Figure S20A), compared to the stationary phase. However, the exponential phase showed progressively decreased sequencing depth toward the DNA replication terminus (*ter*), compared to the stationary phase (upstream: R = 0.79; downstream: R = −0.88; *p* < 0.001; Figure S3A). DNA read start positions were enriched around the *oriC* region in the exponential phase, while fewer read start positions were seen around the *ter* region (*p* < 0.001; Figure 2B, Figures S3B and S20A). In comparison, the distribution of read start sites was relatively even across the genome in the stationary phase. Similar sequencing depth and read start site distribution patterns were seen in *L. acidophilus* (*p* < 0.05; Figures S8B, S9 and S20B). These results indicate that the frequent DNA replication initiation results in disproportionately more DNA fragments around *oriC* (which can be proportionately quantified using Nanopore sequencing) in both *E. coli* and *L. acidophilus*. This potentially resulted from the overlapping replication cycles in *E. coli* [18,19]; however, the reason for this pattern in *L. acidophilus* is unclear.

### 2.4. De Novo DNA Methylation Motif Identification

The potential DNA methylation motifs were first examined by identifying related DNA methylases through genome annotation. In *E. coli*, six potential DNA methylation motifs were identified, while no motif was discovered in *L. acidophilus* (Table S8).

De novo DNA methylation motif identification across bacterial genomes was performed using both Modkit and Snappy [20]. Each program was run twice, once with the NCBI RefSeq genome, and once with the assembled genome as a reference. The results were highly similar regardless of the reference genome used (Tables S9 and S10). Although not all potential motifs were identified, Snappy [20] showed the highest number of methylation motifs and the most consistent identification pattern across samples in *E. coli* (Figure S4; Table S10). In addition, two distinct m4C DNA methylation motifs (5′-GATCNT^m4^C(V)-3′ and 5′-GATCNNT^m4^C-3′; Tables S9 and S10) were discovered by both Modkit and Snappy [20]. In *L. acidophilus*, the 5′-GC^m6^ATC-3′ motif was detected (Figure S10; Tables S9 and S10). In summary, Snappy [20] exhibited the most consistent performance in motif identification and identified the largest number of motifs.

Motifs identified by the two tools across samples were used for subsequent DNA methylation analysis. After validation, four motifs (5′-G^m6^ATC-3′, 5′-C^m5^CWGG-3′, 5′-GATCNT^m4^C(V)-3′, 5′-GC^m6^AGNNNNNTGT-3′) in *E. coli* and one motif (5′-GC^m6^ATC-3′) in *L. acidophilus* met these criteria. While m4C motifs (e.g., 5′-GATCNT^m4^C(V)-3′) were identified in *E. coli*, they significantly overlap with the known m6A motif (5′-G^m6^ATC-3′), raising the possibility of false-positive basecalling artifacts. Therefore, m4C is not included or discussed in subsequent analyses to enable reliable biological inference. We selected two common motifs from *E. coli* (5′-G^m6^ATC-3′ and 5′-C^m5^CWGG-3′) and one motif from *L. acidophilus* (5′-GC^m6^ATC-3′) for DNA methylation level comparison. Selected motifs were defined for DNA methylation calling on both strands of the genome. Subsequently, we refer to the analysis of the methylation within these motifs as a motif-based approach.

### 2.5. Lower DNA Methylation Levels During the Exponential Phase

Active DNA replication initiation during the exponential phase can lead to higher proportions of unmethylated daughter strands. Because of the presence of a larger proportion of these unmethylated daughter strands, we hypothesized that the genome-level and regional DNA methylation levels would be lower during the exponential phase compared to the stationary phase. The genome-level methylation proportions of selected motifs (*E. coli*: 5′-G^m6^ATC-3′ and 5′-C^m5^CWGG-3′; *L. acidophilus:* 5′-GC^m6^ATC-3′) between the exponential and stationary phases were first compared. Each program was run twice, once with the NCBI RefSeq genome, and once with the assembled genome as a reference. All motifs showed significantly lower methylation levels during the exponential phase in both bacterial species, compared to the stationary phase (*p* < 0.05; Figures S5A and S11A). In *E. coli*, principal component analysis (PCA) of the methylation proportion within each motif site separated samples based on growth phase, with 48.77% (RefSeq genome) and 49.59% (assembled genome) DNA methylation variance on the first principal component (Figures S5B and S21C). In *L. acidophilus*, the DNA methylation variation also demonstrated 37.31% (RefSeq genome) and 36.81% (assembled genome) DNA methylation variance on the first principal component (Figures S11B and S21D).

In addition to the common DNA methylation motifs (e.g., 5′-G^m6^ATC-3′ and 5′-C^m5^CWGG-3′), rare motifs can also exist in bacterial genomes. These rare motifs with low occurrence or minimal methylation levels are likely undetectable when using motif-based approaches, leading to missing DNA methylation information. Therefore, we also performed DNA methylation calling in each position on both strands of the genome without specifying DNA methylation motifs, defined as a locus-based approach.

**Figure 3:**
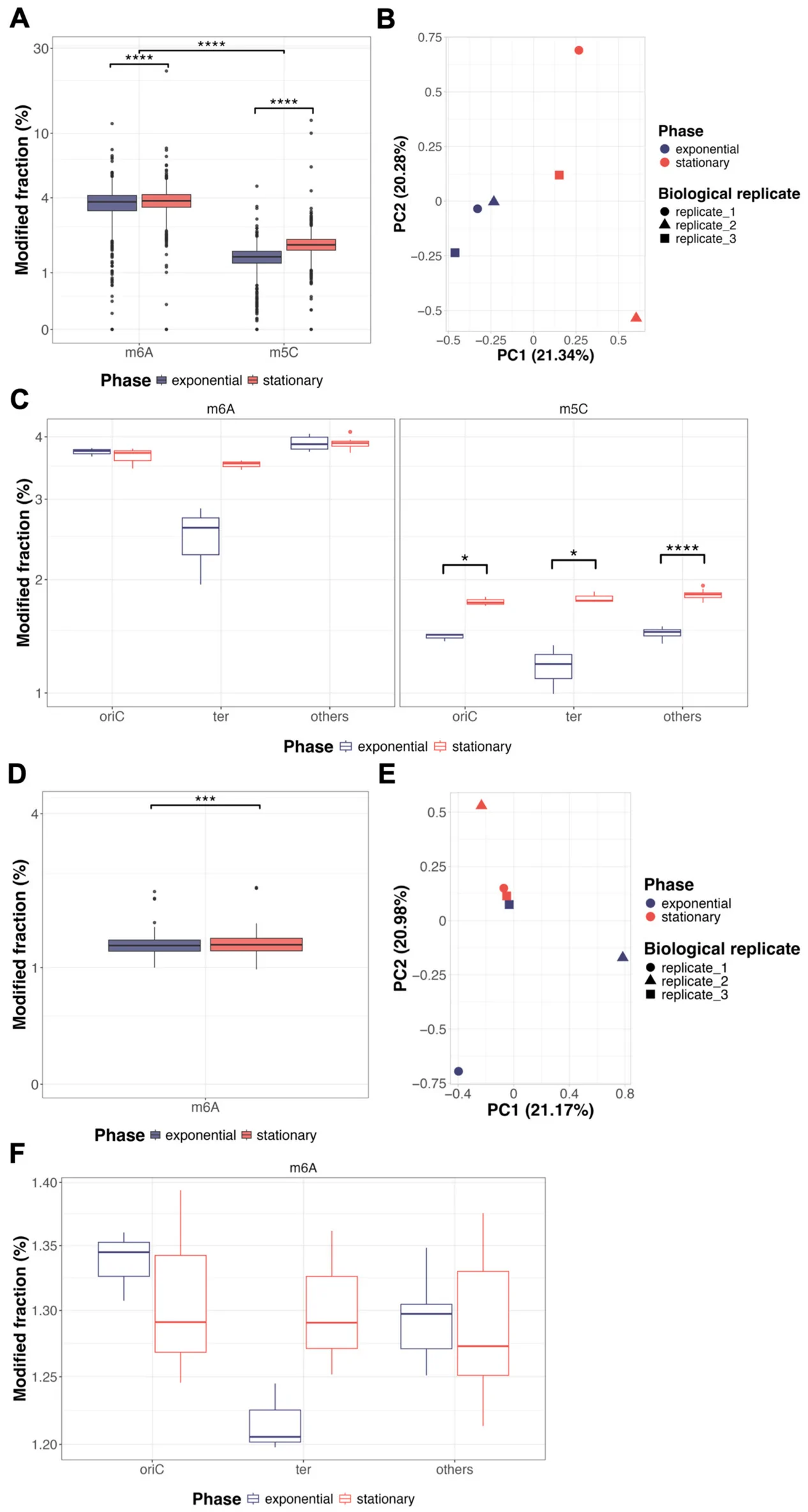
Regional and localized DNA methylation level analyses of *Escherichia coli* (**A**–**C**) and *Lactobacillus acidophilus* (**D**–**F**) using the locus-based approach. (**A**) DNA methylation levels of each 10 kb segment in two DNA methylation forms. (**B**) Principal component analysis on DNA methylation proportions at genomic loci in selected DNA methylation forms (N6-methyladenine and 5-methylcytosine for *Escherichia coli*; N6-methyladenine for *Lactobacillus acidophilus*). (**C**) DNA methylation levels within the DNA replication origin and terminus 100 kb flanking regions, as well as the regions outside these windows, between the exponential and stationary phases. Analysis here only included replicates 1, 2, and 3 with DNA and RNA sequencing data in *E. coli*. The RefSeq genomes (GCF_000008865 for *E. coli*; GCF_003047065.1 for *L. acidophilus*) were used for the analysis. A paired *t*-test was used to compare the modified fraction between different phases, while an unpaired *t*-test was used to compare different DNA methylation forms. A paired *t*-test was used to compare the modified fraction of DNA replication origin, terminus and the other segments between different phases. *ter*: DNA replication terminus 100 kb flanking region. *oriC*: DNA replication origin 100 kb flanking region. Others: regions outside the DNA replication origin and terminus 100 kb flanking regions. m6A: N6-methyladenine. m5C: 5-methylcytosine. *p*-values < 0.05: *; *p*-values < 0.001: *** or ****.

Using the methylation level on each genomic locus, the methylation levels of each 10 kb genome window were calculated to compare the DNA methylation profile between the exponential and stationary phases. Each program was run twice, once with the NCBI RefSeq genome, and once with the assembled genome as a reference. All selected methylation types showed significantly lower methylation levels in the exponential phase compared to the stationary phase (Figure 3A,D) in both *E. coli* (*p* < 0.01) and *L. acidophilus* (*p* < 0.001). In *E. coli*, m6A demonstrated the highest methylated level, followed by m5C. The matrix of DNA methylation levels at each genomic position was also used for PCA to visualize the localized variance. The first principal component separated *E. coli* samples based on growth phase and accounted for 21.34% (RefSeq genome) and 21.23% (assembled genome) of the variation in methylation level (Figure 3B and Figure S21A). Likewise, the first principal component in the *L. acidophilus* data accounted for 21.17% (RefSeq genome) or 20.65% (assembled genome) of the variation in methylation level and clearly separated the exponential and stationary phases (Figure 3E and Figure S21B).

### 2.6. Lower DNA Methylation Levels Around the DNA Replication Terminus

During the bacterial exponential phase, DNA strands are actively unwound for replication, and newly synthesized DNA strands are likely captured before being fully methylated. However, uneven sequencing depth across genomes, particularly around *oriC* and *ter* regions, is observed during the exponential phase. Because DNA methylation characterization relies on the sequenced DNA strands, we hypothesized that these sequencing depth differences could affect the methylation characterization around the *oriC* or *ter* region, potentially introducing bias to the DNA methylation distribution across the genome.

Using each genomic locus, DNA methylation levels within 200 kb of the *oriC* or *ter*, and other segments (i.e., outside the *oriC* and *ter* regions) were compared between the stationary and exponential phases. In *E. coli*, we observed a consistent reduction in m5C methylation in the exponential phase compared to the stationary phase in all three regions (*p* < 0.05; Figure 3C). However, for m6A, there was no evidence of changes in methylation levels in either the *oriC* region or the genomic regions outside *ter* and *oriC*. Likewise, the m6A levels were lower within the *ter* region in *L. acidophilus* during the exponential phase (*p* > 0.05; Figure 3F).

**Figure 4:**
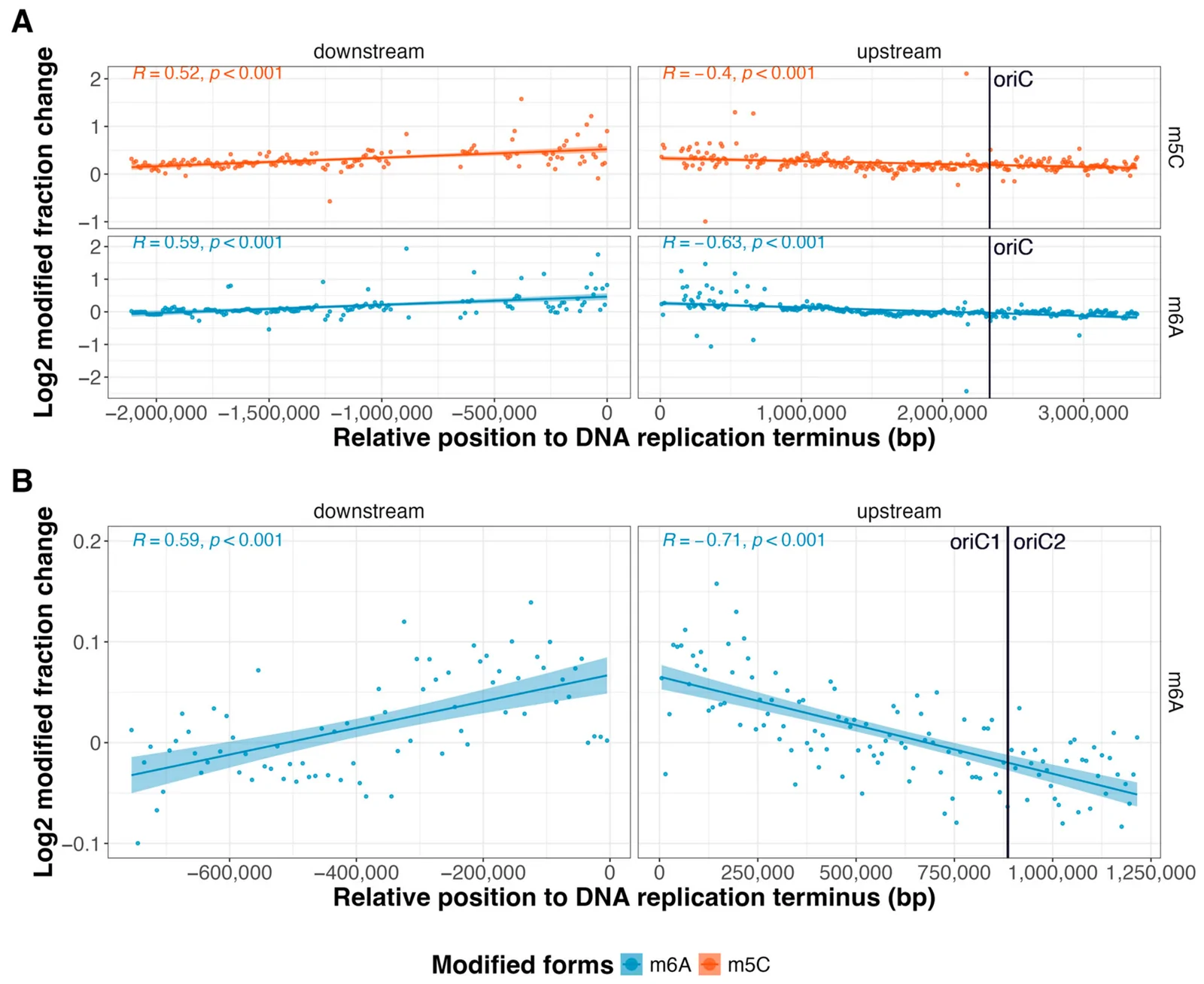
DNA methylation level changes across the genome of *Escherichia coli* (**A**) and *Lactobacillus acidophilus* (**B**) using the locus-based approach. DNA methylation level changes in each 10 kb window between the exponential and stationary phases around the DNA replication terminus. Analysis here only included replicates 1, 2, and 3 with DNA and RNA sequencing data in *E. coli*. The RefSeq genomes (GCF_000008865 for *E. coli*; GCF_003047065.1 for *L. acidophilus*) were used for the analysis. Only the data from the chromosomes NC_002695.2 (*E. coli*) and NZ_CP139575.1 (*L. acidophilus*) were included. The log2-transformed modified fraction change is the base-2 logarithm of the stationary value relative to the exponential value. The Spearman correlation coefficient was calculated to estimate the relation between distance to the DNA replication terminus and log2-transformed modified fraction change. *oriC*: DNA replication origin. m6A: N6-methyladenine. m5C: 5-methylcytosine.

Using each genomic locus, the mean DNA methylation level changes between the exponential and stationary phases of each 10 kb window across the genome were compared, without accounting for spatial autocorrelation between adjacent windows. Each program was run twice, once with the NCBI RefSeq genome (435 windows for *E. coli*; 198 windows for *L. acidophilus*), and once with the assembled genome as a reference (479 windows for *E. coli*; 198 windows for *L. acidophilus*). Using the RefSeq genome, the *ter* was used as a reference point to define upstream and downstream regions (Figure 4A,B). During the exponential phase of *E. coli*, the DNA modification levels in two DNA methylation forms decreased toward *ter* (*p* < 0.001; Figure 4A), compared to the stationary phase. The level of m6A was the most affected by the distance to *ter* (upstream: R = 0.59; downstream: R = −0.63), followed by m5C (upstream: R = 0.52; downstream: R = −0.40). A similar reduction pattern in m6A towards *ter* was seen in *L. acidophilus* (Figure 4B). For assembled genomes, although no *ter* positions were predicted, methylation reduction patterns were also observed with increasing distance from *oriC* in both *E. coli* and *L. acidophilus* (Figure S22).

A linear mixed model was used to further evaluate the effects of sequencing depth and growth phases on DNA methylation. Each program was run twice, once with the NCBI RefSeq genome, and once with the assembled genome as a reference. Based on the findings above, we hypothesize that: (1) the sequencing depth does not affect the percentage of methylated DNA; and that (2) the growth phase transition influences DNA methylation levels in *E. coli*, where DNA replication is more active during the exponential phase compared to *L. acidophilus*. However, we observed that sequencing depth had a significant effect on DNA methylation detection in both species (*p* < 0.001; Table S18). DNA methylation levels were also significantly affected by growth phase transition in *E. coli* (*p* < 0.001; Table S18), and this pattern was also observed in *L. acidophilus* (*p* < 0.05). These results suggested that both growth phase transition and sequencing depth were factors affecting DNA methylation levels.

### 2.7. Consistent Sequencing Depth and DNA Methylation Distribution Patterns

To validate the observed sequencing depth and DNA methylation distribution patterns, an independent *E. coli* dataset was generated. The analysis followed the pipeline used for the original dataset. Each program was run twice, once with the NCBI RefSeq genome, and once with the assembled genome as a reference. Consistent with the original findings, sequencing depth decreased around *ter* during the exponential phase compared to the stationary phase, while the opposite pattern was observed around *oriC* (Figure S18A). Similarly, although the sequencing coverage increased to 120× in the validation dataset, lower DNA methylation levels, especially m6A, were still observed around *ter* during the exponential phase, compared to the stationary phase (*p* < 0.05; Figure S18B). In addition, DNA methylation motifs identified in the validation dataset (Table S37) were consistent with the original findings. These results demonstrate the robustness of the observed sequencing depth and DNA methylation distribution patterns.

### 2.8. Differentially Methylated and Expressed Genes Are Associated with Physiological Changes

We first compared the efficiencies of locus-based and motif-based approaches in differential methylation position identification between growth phases. Each program was run twice, once with the NCBI RefSeq genome, and once with the assembled genome as a reference. In *E. coli*, the motif-based method constituted a subset of the locus-based profile and reflected 20.9% to 72.9% (RefSeq genome) or 19.9% to 84.7% (assembled genome) of the differentially methylated positions in the locus-based method (*p* < 0.001; Figures S7 and S23A–C). The overlapping proportions increased with the motif numbers. In *L. acidophilus*, the locus-based protocol also identified more differentially methylated sites (*p* < 0.001; Figures S13 and S23D). These results indicated that the locus-based protocol was more efficient in capturing differentially methylated sites in the genome.

**Figure 5:**
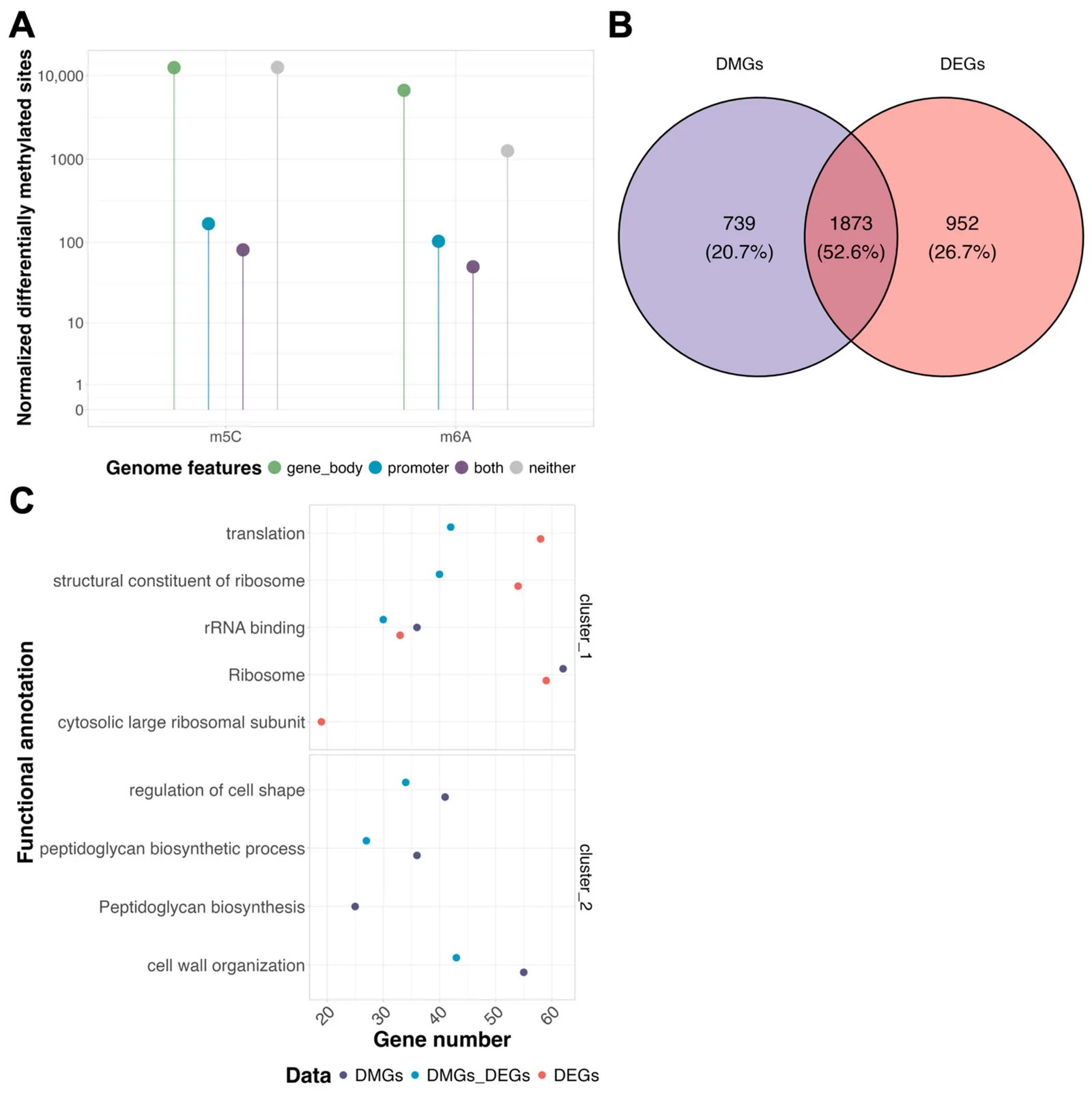
Locus-based differential methylation, differential expression, and functional annotation analyses in *Escherichia coli*. (**A**) Differentially methylated position distribution. (**B**) The number of shared genes with differential methylation and expression. (**C**) Functional annotation clustering of genes with differential methylation and expression. Analysis here only included replicates 1, 2, and 3 with DNA and RNA sequencing data in *E. coli*. The RefSeq genome (GCF_000008865) was used for the analysis. The promoter region was defined as the −35 to −7 frame upstream of the open reading frame start site, while the gene body referred to the region from the open reading frame start to the end site. The differentially methylated sites were normalized by the background GC content. The DNA methylation profile in the Venn graph included differentially methylated genes across three DNA methylation forms. Fisher’s Exact Test was performed to evaluate the gene overlap significance between the DNA and RNA datasets. The functional annotation analysis used the Gene Ontology and KEGG databases with *E. coli* O157:H7 str. Sakai as a background. The analysis only included the terms with the modified Fisher Exact *p*-value equal to or less than 0.05. m6A: N6-methyladenine. m5C: 5-methylcytosine. DMGs: differentially methylated genes. DEGs: differentially expressed genes. DMGs_DEGs: genes that were both differentially methylated and expressed.

DNA methylation is considered a gene expression regulator, reflected by the differential methylation levels on promoters or gene bodies. Therefore, the differential methylation positions identified by the locus-based method were located in promoters or gene bodies. The differentially methylated sites were normalized by the GC content. Despite being the least abundant methylation form in prokaryotes, m5C had the most number of differentially methylated sites in gene bodies and promoters in *E. coli* (Figure 5A and Figure S24). As the most abundant methylation form in prokaryotes, m6A exhibited the highest number of differentially methylated sites within gene bodies in *L. acidophilus* (Figure S14).

To examine the association between methylation and gene expression, the number of genes with differential methylation and expression was compared. In *E. coli*, 2825 (RefSeq genome) or 3342 (assembled genome) differentially expressed genes were identified between the exponential and stationary phases. A total of 71.71% (RefSeq genome) or 71.32% (assembled genome) of the differentially methylated genes were differentially expressed between the two growth phases (*p* < 0.001; Figure 5B and Figure S25). In contrast, the differentially methylated genes from the motif-based approach reflected fewer differentially expressed genes, especially when only 5′-G^m6^ATC-3′ was selected (*p* < 0.001; Figure S16A). These results indicated that the differentially methylated gene profile from the locus-based approach was reflective of the overall differentially expressed gene profile.

Generally, bacteria in the exponential phase grow rapidly because of the rich nutrients, while a slow-growing speed is seen in the stationary phase due to limited nutrients. Therefore, the functions of the differentially methylated and expressed genes were further examined to evaluate whether they were associated with the starvation response during the stationary phase. In practice, using a RefSeq reference genome is preferable due to its computational efficiency, streamlined functional analysis, and high-quality annotations. In addition, our findings demonstrated that using a RefSeq genome provided biological inferences comparable to those using an assembled genome, due to the high genome similarities. Therefore, subsequent analyses were performed using the RefSeq-based results.

In *E. coli*, the differentially methylated and expressed genes formed two clusters with similar functional enrichment (Figure 5C; Table S26). In cluster one, genes were significantly associated with translation activities (*p* < 0.05), such as translation and rRNA binding. In cluster two, the genes were related to cell membrane synthesis (*p* < 0.05), such as the regulation of cell shape and peptidoglycan biosynthesis. In *L. acidophilus*, the functions of the differentially methylated genes are mostly related to the carbohydrate catabolic process and nucleic acid binding (*p* < 0.05; Table S27). These results indicate that bacteria may respond to nutritional limitation during the stationary phase by tuning their metabolic and reproductive activities.

### 2.9. DNA Methylation Showed a Weak Linear Correlation with Gene Expression

DNA methylation on promoter regions and gene bodies can affect gene regulation [21]. Therefore, a linear model was applied to the *E. coli* data to examine the correlation between DNA methylation levels (promoter and gene body regions) and gene expression. The analyses were performed using the RefSeq-based results.

To capture the most information on DNA methylation and gene expression, regardless of the specific motifs that they exist in, all differentially methylated positions within the promoters and gene bodies on both strands of the genome were included in the analysis. Only the genes showing both differential methylation and expression were used in the analysis. Growth phases and replicate effects were included in the model.

**Figure 6:**
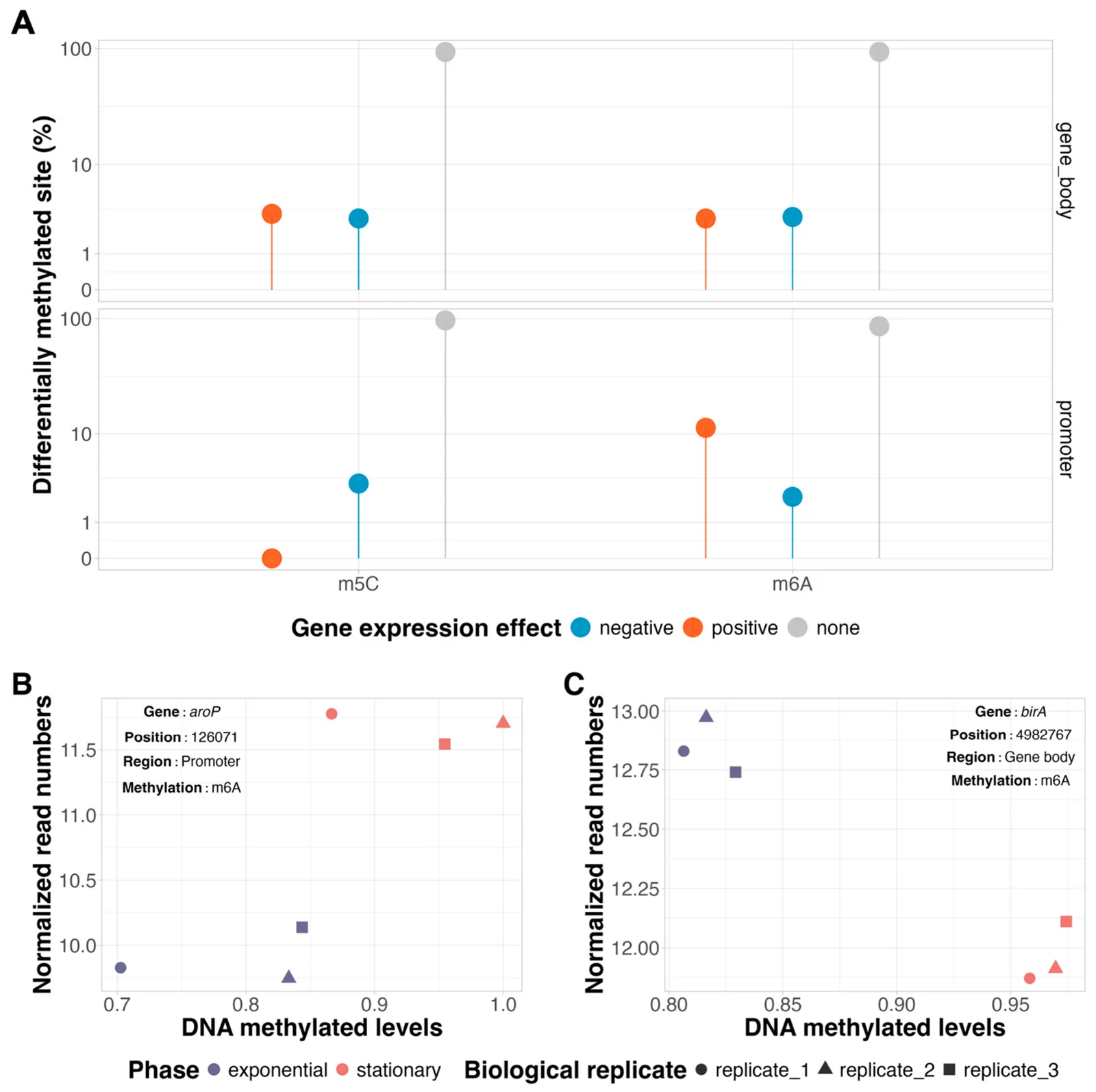
Linear correlation analysis of *Escherichia coli* between the locus-based DNA methylation and gene expression. (**A**) The proportions of differentially methylated genomic sites by gene expression effects. DNA methylation and gene expression pattern of (**B**) *aroP* and (**C**) *birA* genes. Analysis here only included replicates 1, 2, and 3 with DNA and RNA sequencing data in *E. coli*. The RefSeq genome (GCF_000008865) was used for the analysis. The promoter region was defined as the −35 to −7 frame upstream of the open reading frame start site, while the gene body referred to the region from the open reading frame start to the end site. m6A: N6-methyladenine. m5C: 5-methylcytosine.

In both promoter and gene body regions, the DNA methylation level of over 92% of differentially methylated sites in three methylation forms showed a weak linear correlation with gene expression (*p* > 0.05; Figure 6A). DNA methylation levels at several loci within the gene body showed a strong linear correlation with the expression of the corresponding gene (*p* < 0.05; Figure 6A). For example, DNA methylation levels at the genomic position 4,982,767 of the *birA* gene body (Gene product: Bifunctional ligase/repressor BirA) were negatively correlated with BirA transcript expression (Figure 6C). Around 7% of differentially methylated sites in the defined promoter regions showed strong effects (*p* < 0.05). For instance, the expression of AroP (Aromatic amino acid transport protein) was positively correlated with the DNA methylation level on its defined promoter region position 126,071 (Figure 6B). Functional annotation results showed that most genes with expression affected by DNA methylation were associated with GTPase activity and guanosine tetraphosphate binding (*p* < 0.01; Table S32). In summary, the methylation levels in most differentially methylated sites (defined promoters and gene bodies) showed a weak linear correlation with the corresponding gene’s expression.

## 3. Discussion

This study examined the genome-wide DNA methylation and localized DNA fragment density differences between the exponential and stationary phases in *E. coli* and *L. acidophilus*. In both bacterial species, sequencing depth was higher around *oriC* in the exponential phase compared to the stationary phase, while lower sequencing depths were observed near *ter*. We identified variation in m6A and m5C DNA methylation associated with the growth cycle of prokaryotic cells. Regions surrounding *ter* showed consistently lower methylation levels during the exponential phase, particularly for m5C. The locus-based DNA methylation calling showed a higher efficiency in differentially methylated site detection, compared to the motif-based one. Although only a fraction of differentially methylated sites in the defined promoters and gene bodies of *E. coli* were significantly associated with gene expression, the locus-based differentially methylated gene profile significantly reflected the differentially expressed gene profile in *E. coli*. These genes were closely related to translation and cell membrane synthesis activities. In *L. acidophilus*, the functions of differentially methylated genes were mostly related to nucleic acid binding and carbohydrate catabolic processes. Our findings indicated that the bacterial DNA methylation pattern can be affected by growth phase transition, and DNA methylation could be used as a simplified indicator of microbial activity.

Fast-growing bacteria, such as *E. coli*, can undergo overlapping replication cycles, where a new replication round is initiated before the previous round is terminated, under rich nutrients [18,19]. We expect that this phenomenon would lead to an increase in the DNA fragment density near *oriC*, compared to the rest of the genome. Consistent with this theory, our study found an increased frequency of read start positions and higher sequencing depth around the *oriC* region of *E. coli* during the exponential phase, and lower coverage near *ter*. Despite the lack of studies demonstrating overlapping replication cycles in *L. acidophilus*, it also showed a similar sequencing depth and read start distribution pattern during the exponential phase, which can be partially due to active DNA replication activity [22]. Consistent with this finding, previous studies have found a lower read number around *ter* in the exponential phase of *E. coli* compared to *oriC*, while this pattern was not observed in the stationary phase [23,24]. In summary, the active cell division during the exponential phase is accompanied by elevated DNA replication activity, which may affect the sequence distribution across the genome after DNA sequencing.

The uneven sequencing coverage in the exponential phase may make sequencing data from this stage unsuitable for common analyses. Genome assembly and variant calling accuracy and quality are both affected by the sequencing coverage uniformity [25,26]. As such, studies using data from the exponential phase may have a bias in the error location due to the low coverage at the terminus. This is also true for methylation analyses, which rely on minimum depths to accurately estimate the percentage of methylated bases. In our study, we determined the sufficient sequencing coverages for DNA methylation characterization; however, our results also suggested that a higher sequencing coverage (>100×) may be required for DNA methylation calling for samples from the bacterial exponential phase. Therefore, it is recommended that at least twice as much data as needed for studies using data from the exponential phase, to account for the two times lower coverage near *ter*.

DNA methylation motif identification and genome-level motif frequencies, especially m6A-related motifs, are widely used for capturing bacterial DNA methylome differences among environments, e.g., [13,14,15,16,27]. In prokaryotes, m6A is generally regarded as the prevalent DNA methylation [4]. Moreover, frequent phage-mediated genetic exchanges drive the prokaryotic DNA methylome variation from diverse environments, reflected in DNA methylation motifs and DNA methylase genes [28,29,30]. However, instead of m6A, we found that m5C had the most differentially methylated sites within gene bodies and promoters in *E coli* between growth phases. A previous report also showed that the abundances of different DNA methylation forms varied across different bacterial species [31]. Similarly, another study using Oxford Nanopore sequencing found that m4C and m5C were more dominant in *Klebsiella pneumoniae*, a Gram-negative bacterial species [13]. One potential reason for this is that motif-based analysis is limited to genetic (e.g., genetic variation) or background (e.g., sequencing depths) factors, and they may fail to identify rare motifs that are difficult to detect, leading to missing DNA methylation information. In our study, we found that the motif-based approach can only capture a fraction of the differentially methylated positions of the locus-based protocol. In addition, the motif-based approach reflected smaller proportions of the differentially expressed genes, especially when only one motif was included. Although incorporating more motifs can improve position and gene identification, the locus-based protocol is comparatively more efficient. These findings indicate that focusing only on the most common methylation form or limited motifs will cause important biological relationships to be missed. This highlights the importance of studying multiple DNA methylation forms and the careful decision on motif-related analysis.

The locus-based, motif-independent analysis pipeline developed in this manuscript can also provide deeper biological insights into data integration. To our knowledge, this is the first application of this approach to DNA methylation analysis. Previous studies have used segmental or genome-level DNA methylation fractions of specific motifs to visualize bacterial DNA methylation variation under different environments [15] or physiological states [32]. However, DNA methylation mediates genome defense [1] or gene regulation functions [3] primarily by methylating specific genomic loci rather than broad genomic regions. Therefore, when integrating other omics data (e.g., transcriptomics and proteomics), regional DNA methylation fractions may fail to reveal the biological meaning behind the locus-level DNA methylation variation. These limitations also apply to analyses only using specific motifs or methylation types, as bacteria utilize diverse DNA methylation systems to regulate cellular activity. Overall, using a locus-based, motif-independent analysis pipeline can be more efficient in data integration.

The exponential phase of *E. coli* and *L. acidophilus* showed significantly lower levels of three DNA methylation forms (m6A or m5C). The selected motifs (e.g., 5′-G^m6^ATC-3′ and 5′-C^m5^CWGG-3′) during the exponential phase also showed significantly lower methylation levels. Consistent with our results, a previous study found that m5C levels increased in *E. coli* from the exponential phase, through the late-exponential and transition-to-stationary phases, reaching the highest levels in the stationary phase [24]. Likewise, another study using *Pseudomonas syringae* observed that higher m6A levels were found in the stationary phase compared with the exponential phase [33]. Several studies have indicated possible reasons for this phase-specific DNA methylation level. For example, it was proposed that the lower m5C DNA methylation in the 5′-C^m5^CWGG-3′ motifs was to minimize deleterious mutations in *E. coli* during the exponential phase, where the Very Short Patch (VSP) repair system is not expressed [24]. In addition, the competition of DNA-binding proteins with DNA methylases may also contribute to the lower methylation in the exponential phase [34,35]. Taken together, these findings support the hypothesis that most DNA methylation is downregulated in actively dividing prokaryotic cells, although the mechanism for this is not necessarily clear.

Lower methylation levels were observed around *ter* during the exponential phase in our study. These lower methylation levels may result from the methylation site blocking due to the DNA replication termination protein binding. For instance, during DNA replication termination in *E. coli*, Tus proteins can interact with *ter* sequences distributed around the DNA replication terminus, forming a Tus-*ter* complex for replisome trapping [36]. The Tus protein can protect its interacting DNA motif from being methylated [36], potentially causing lower DNA methylation levels around *ter*. However, DNA replication can also be terminated through replisome fork fusion without Tus proteins [37], indicating that methylation motifs were not completely impeded by Tus proteins. Therefore, the Tus-*ter* complex cannot fully explain the systematically lower DNA methylation levels surrounding the *ter* region and the real mechanism behind the regionally lower DNA methylation in the exponential phase needs further investigation.

DNA methylation is generally considered a gene regulation tool in organisms [5]. However, methylation levels at only a fraction of differentially methylated positions in the gene bodies and defined promoters showed a strong linear correlation with gene expression levels. Similarly, studies using *Ralstonia pseudosolanacearum* and *Salmonella enterica* demonstrated that only a fraction of differentially methylated positions or genes showed a significant association with gene expression [14,38]. Due to limited research on bacterial gene promoters, the promoters used in our study may not represent the true promoter regions, which could affect the identification of differentially methylated positions with significant effects. The bulk methylation and gene expression profile in this study also limited the resolution of this methylation-expression correlation. On the other hand, our study only performed the correlation analysis for a single differentially methylated position, while DNA methylation may execute gene regulation through the methylation patterns of several positions. For example, in *S. enterica*, the DNA methylation on the first and third 5′-G^m6^ATC-3′ motifs in the *opvAB* regulatory region allowed the expression of OpvA and OpvB, while the expression was switched off when the second and fourth motifs were methylated [6]. Furthermore, the small proportion of associated methylated positions may indicate that other molecular regulatory mechanisms, such as the sigma factor RpoS [39], non-coding RNA [40], or DNA phosphorothioation [41], could contribute to gene expression. Therefore, future research could study bacterial regulatory elements and assess how different DNA methylation patterns within elements affect gene expression at the single-cell level. In summary, DNA methylation is related to gene expression in bacteria; however, the complexity of gene regulation networks in bacteria also suggests that further studies are required to enhance the understanding of bacterial DNA methylation in gene regulation.

Despite the weak linear correlation between DNA methylation levels and gene expression, almost 72% of differentially methylated genes in *E. coli* were differentially expressed, indicating that the DNA methylation pattern can broadly represent the transcriptomic profile. This suggests that DNA methylation profiling can be an efficient and cost-effective method to evaluate bacterial activities, considering the relatively higher cost and complexity of generating prokaryotic RNA sequencing data. While transcriptomic data remains critical for comprehensively characterizing bacterial responses to environmental perturbations, in many cases, the cost-effective combination of genome sequence characterization and methylation quantification when using long-read sequencing is an appropriate methodological choice. Specifically, the locus-based DNA methylation approach developed in this study may provide an additional omics layer for assessing microbial activity in microbiome samples. However, challenges, including strain heterogeneity and insufficient sequencing depth for low-abundance species, need to be addressed before application.

Generally, nutrients are not replenished in a batch culture system, contributing to nutrient depletion during long-term bacterial cultivation. Depleted nutrition becomes apparent during the stationary phase, which requires bacteria to undergo adaptations, such as metabolic and morphological changes [9,42,43]. Consistent with this, the differentially methylated and expressed genes we observed were closely related to physiological changes: in *E. coli*, functions related to translation and cytoplasmic synthesis were enriched; in *L. acidophilus*, genes related to the carbohydrate catabolic process were differentially methylated. However, functional enrichment in *L. acidophilus* was weaker, which may reflect its smaller gene repertoire and limit the biological interpretation of these findings. Similar results have been observed in other studies, e.g., genes involved in carbohydrate metabolism were differentially expressed between the exponential and stationary phases in *Lacticaseibacillus rhamnosus* [43], while the *rpoS* gene in *E. coli* (which regulates morphology and metabolic activities under stationary phase or starvation conditions [44,45]) showed higher methylation in the stationary phase in Kahramanoglou et al. [24] and our study. These functional changes indicate that bacteria may modulate their metabolic or reproductive activity in response to starvation. Therefore, our findings suggest that the DNA methylation profile can broadly reflect the bacterial physiological changes during the growth phase transition.

Here we have only used two bacterial species for analysis. These two species do not cover the full extent of diverse characteristics, such as growth rates, GC contents, DNA methylases, and gene regulators within prokaryotes. Therefore, it is prudent for future work to evaluate these patterns in different species and in different environments. Furthermore, future studies should develop cross-species methylation models for robust DNA methylation analysis and integrate orthogonal approaches, such as SMRT sequencing, to validate methylation signals. Still, the similarity in methylation patterns between the two species used here suggests that the patterns observed here are potentially common within prokaryotes. Overall, it is clear that the examination of all methylatable loci, and the multiple forms of methylation, is critical to understanding the epigenetic landscape of prokaryotes. With advances in direct detection of methylation through sequencing, it is now cost-effective to study prokaryotic methylation and its link to key biological processes such as cellular division.

## 4. Materials and Methods

### 4.1. Bacterial Strains and Growth Media

Two bacterial species, *Escherichia coli* (isolated from chicken fecal samples) and *Lactobacillus acidophilus* DSM 20079, were utilized in this study. The *E. coli* is generally considered fast-growing, while *L. acidophilus* is slow-growing (Figures S1 and S2); this contrast enabled the examination of localized DNA fragment density and methylation patterns in bacteria with different growth rates. Both bacteria were cryopreserved in 2 mL tubes with 25% glycerol at −80 °C. Revival of *E. coli* was performed by streaking the bacterial stock on a Luria–Bertani (LB) agar and incubation at 37 °C aerobically for 15 h. For culture in liquid media, a single *E. coli* colony was picked from the LB agar and inoculated into a 250 mL Erlenmeyer Flask with 75 mL LB broth. Revival of *L. acidophilus* was performed by streaking the bacterial stock on a De Man–Rogosa–Sharpe (MRS) agar and incubation at 37 °C aerobically for 39 h. Four single *L. acidophilus* colonies were picked from the MRS agar and inoculated into a 250 mL Erlenmeyer Flask with 75 mL MRS broth.

### 4.2. Bacterial Growth Curves

The *E. coli* was cultured in 75 mL LB broth at 37 °C under 200 rpm, in three biological replicates. The *E. coli* suspension was collected every 1.5 h during the first 12 h and 3 h over the subsequent 12 h. The *L. acidophilus* was grown in 75 mL MRS broth at 37 °C statically (no shaking), in three biological replicates. The *L. acidophilus* suspension was collected every 3 h during the first 24 h and 6 h over the next 24 h. The colony-forming unit (CFU) of each timepoint was calculated by counting the colonies of the agar plates inoculated with serially diluted bacterial suspensions. The OD600 value of each timepoint was measured with the corresponding sterile broth as a blank. The measured OD600 values were imported into RStudio to generate a growth curve. The OD600 values at every 0.5 h of each culture were imputed using Gompertz statistical models with observed OD600 values as input. The imputed values were used as references to determine the sample collection times.

### 4.3. Sample Collection and Sequencing

After determining the growth curves, new liquid cultures were set up, and bacterial suspensions were collected at the exponential and stationary phases (Table 1). The *E. coli* culture included five biological replicates (three replicates containing RNA sequencing data; Table 1), while the *L. acidophilus* culture consisted of three biological replicates. Bacterial suspensions from the exponential and stationary phases for DNA and RNA extraction were collected from the same biological replicate without additional sampling.

For DNA extraction, bacterial suspensions were collected and centrifuged at 16,000× *g* for 5 min, followed by removing the supernatant. The *E. coli* DNA was extracted from the pellets using the Puregene Kit (QIAGEN, Hilden, Germany) with the manufacturer’s instructions. The *L. acidophilus* DNA was extracted using the Puregene Kit (QIAGEN, Germany) with slight modifications for Gram-positive bacteria. In brief, the cell lysis was conducted with lysozyme (100 mg/μL) and Proteinase K at 37 °C for 1 h, followed by a 1 h incubation at 56 °C. The DNA library was prepared with the Native Barcoding Kit 24 V14 (ONT, Oxford, UK) or Native Barcoding Kit 96 V14 (ONT, Oxford, UK) using the extracted DNA. The DNA library was loaded on a FLO-PRO114M (R10.4.1) Flow Cell, followed by sequencing on the PromethION 2 Solo (ONT, Oxford, UK) with at least six samples from each bacterial species reaching 100× coverage.

For RNA extraction, one volume of bacterial suspension was directly added to two volumes of RNAprotect^®^ Bacteria Reagent (QIAGEN, Hilden, Germany) for RNA stabilization. The RNA extraction was performed with the RNeasy^®^ Protect Bacteria Mini Kit (QIAGEN, Hilden, Germany) under the manufacturer’s instructions. RNA samples with a RIN value of 7 or higher were used for RNA sequencing. The RNA library was prepared following the instructions for Illumina Stranded Total RNA Prep with Ribo-Zero Plus (Illumina, San Diego, CA, USA). The RNA library was loaded on a NovaSeq X 10B flow cell, followed by sequencing on the NovaSeq X Plus (Illumina, San Diego, CA, USA) with at least 50M reads for each sample.

To generate a validation dataset, a new LB culture was set up by inoculating *E. coli* (Table 1). The sample collection and DNA extraction were performed following the instructions mentioned above. The DNA library was prepared with the Native Barcoding Kit 96 V14 (ONT, UK) using the extracted DNA following the protocols from the manufacturer with slight modifications. In brief, barcoded samples were heated at 80 °C for 5 min before pooling to minimize the potential barcode cross-contamination. The DNA library was loaded on a FLO-PRO114M (R10.4.1) Flow Cell, followed by sequencing on the PromethION 2 Solo (ONT, UK) with six samples reaching 120× coverage.

### 4.4. Genome Assembly and DNA Methylase Gene Inference

The stationary-phase *E. coli* replicate 2 from the validation dataset and the stationary-phase *L. acidophilus* replicate 3 from the original dataset were used for genome assembly. After basecalling with the super accurate (SUP) model of ONT Dorado v0.8.0, reads shorter than 250 bp were removed using Nanofilt v2.8.0 [46]. Genome assembly was performed using the filtered reads by Flye v2.9.6 [47] with the “--nano-hq” flag, followed by genome polishing using medaka v2.2.0 and the quality evaluation by QUAST v5.3.0 [48]. Prokka v1.15.6 [49] was used to annotate the polished genome. The predicted protein sequences from genome annotation were used to estimate the genome completeness by compleasm v0.2.7 [50]. The genome similarity between the NCBI RefSeq genomes (*E. coli*: GCF_000008865; *L. acidophilus*: GCF_003047065.1) and assembled genomes was estimated using FastANI v1.34 [51].

Gold standard protein sequences (*n* = 3634, accessed 5 March 2026) from REBASE [52] were retrieved and used for database construction by BLAST+ v2.14.1 [53]. The predicted protein sequences were blasted to the constructed protein database using BLAST+ v2.14.1 [53]. The extremely small number of protein sequences in the database can deflate E-values, providing limited discriminatory power for assessing homology. Therefore, sequence identity, query coverage, and bitscore were used as selection criteria instead of E-value. BLAST results with sequence identity ≥ 90% and query coverage ≥ 90% were retained. For query sequences with multiple hits, the top hit with the highest sequence identity and bitscore was retained, followed by DNA methylation motif retrieval on REBASE.

### 4.5. Data Basecalling and Alignment

DNA sequencing data were basecalled using ONT Dorado v0.8.0 under the SUP model, along with characterizing N6-methyladenosine (m6A), N4-methylcytosine (m4C), and 5-methylcytosine (m5C) in DNA samples. Data were aligned to an NCBI RefSeq genome (*E. coli*: GCF_000008865; *L. acidophilus*: GCF_003047065.1) or to the corresponding assembled genome, using minimap2 v2.28 [54] with an extra flag “-y” to preserve all methylation tags. Primary mapped data were extracted from the BAM files from minimap2 using SAMtools v1.13 [55]. Primary mapped reads shorter than 250 bp were removed using Nanofilt v2.8.0 [46], followed by the subsampling to eleven coverages (1×, 10×, 20×, …, 100×) using Rasusa v2.0.0 [56]. Filtered reads were mapped to the reference genome again using minimap2 v2.28 [54] to generate BAM files with only primary mapped reads. Except for sequencing depth determination analysis, the coverage for *E. coli* and *L. acidophilus* datasets was 60× and 100×, respectively. For the *E. coli* validation dataset, each sample was subsampled to 120× coverage.

### 4.6. Sequencing Depth Determination5

To determine the sequencing depth, BAM files with different coverages (1×, 10×, 20×, …, 100×) were used to generate corresponding bedMethyl tables (1×, 10×, 20×, …, 100×) through ONT Modkit v0.4.1, using NCBI RefSeq or assembled genome as a reference. No specific motif was defined during the bedMethyl table generation. All positions in a bedMethyl table, including positions with or without detected modified bases, were utilized in the analysis. For each bedMethyl table (1×, 10×, 20×, …, 100×), the methylation proportion of each locus was calculated by dividing the methylated base number (N_mod_) by the total number of both methylated and unmethylated bases (N_valid_cov_) of each position. Only loci present in all subsampled datasets were included in the analysis. The Pearson correlation coefficients were calculated to evaluate the correlation between different sequencing depths and 100× coverage using the mean methylation proportions.

### 4.7. DNA Length and Localized DNA Fragment Density

The average length and N50 of the primary mapped reads, as well as the read number of each replicate, were calculated using a customized bash script. The Spearman correlation coefficient between the average length and read number was calculated. A paired *t*-test was used to compare the average DNA length and read number between the stationary and exponential phases.

The DNA replication terminus (*ter*) and origin (*oriC*) positions of the NCBI RefSeq genome or assembled genome were predicted using Ori-Finder 2022 [57]. For *E. coli*, the *ter* or *oriC* were defined by the midpoint of the predicted *ter* or *oriC* genomic regions. For *L. acidophilus*, the *ter* was defined by the single predicted *ter* position. We defined the *oriC* or *ter* 100 kb flanking region as the nucleotides 100 kb upstream and 100 kb downstream of *oriC* or *ter*, respectively.

The BAM files with primary mapped reads were converted to BED files using BEDTools v2.30.0 [58], followed by the extraction of the corresponding genomic position aligned to the 5′ end of each DNA read. Specifically, the start position of each read in the BED file was extracted for reads mapped to the forward strand, while the end positions were extracted for reads aligned to the reverse strand. The 5′ end of each DNA read was considered a proxy for the starting position for the newly synthesized DNA strand. The read starting position numbers within the *oriC* and *ter* 100 kb flanking regions, and the remaining genomic regions were calculated. The read starting sites were divided by the region size for normalization. The normalized read starting sites within the 100 kb flanking *oriC* and *ter* regions, as well as the other regions, were compared through an unpaired *t*-test. *p*-value was adjusted by the Holm–Bonferroni method for multiple group comparisons.

The localized DNA fragment density was determined by the sequencing bases at each respective location, assuming no localized bias during sequencing. SAMtools v1.13 was used to calculate the sequencing depth of each position in the genome using the primary mapped data. The sequencing depth of each 10 kb genomic segment was defined as the sum of base numbers at each position within the segment divided by the segment size. The distance to *ter* or *oriC* was defined as the distance between the midpoint of a 10 kb window and *ter* or *oriC*, respectively. The differential sequencing depth of each 10 kb window was calculated by the formula below:

$$S=\log_{2}\;((C+1)/(D+1))$$

where *S* is the differential sequencing depth of a 10 kb window; *C* is the mean sequencing depth from the stationary phase of a 10 kb window; *D* is the mean sequencing depth from the exponential phase of a 10 kb window; two pseudo-counts of 1 are added to the formula to avoid zero values. The Spearman correlation coefficient between the distance to *ter* and the differential sequencing depth was calculated.

### 4.8. DNA Methylation Proportion per Locus

The NCBI RefSeq genome or assembled genome was used as a reference genome for DNA methylation calling. Using the BAM file and corresponding reference genome, DNA methylation was called through ONT Modkit v0.4.1 to generate a bedMethyl table without specifying motifs, defined as a locus-based method. Genomic positions with coverage lower than 20× were removed. Only loci present in both growth phases were included in the comparison. The methylation proportion of each locus was calculated by dividing the methylated base number (N_mod_) by the total number of both methylated and unmethylated bases (N_valid_cov_) of each position in the bedMethyl tables. These methylation proportions were used for PCA of m6A and m5C in *E. coli* and m6A in *L. acidophilus*.

### 4.9. De Novo DNA Methylation Motif Identification

De novo DNA methylation motif discovery across the bacterial genome was performed using Modkit v0.4.1 and Snappy (v2026) [20]. The NCBI RefSeq genome or assembled genome was used as a reference genome for DNA methylation motif identification. Motifs identified by two tools across samples were used for subsequent DNA methylation analysis.

Using the locus-based bedMethyl file and reference genome file as input, ONT Modkit v0.4.1 was applied to identify rare motifs (low occurrence and low methylation levels) with the following settings: the low modification level threshold was 0.01 (--low-thresh 0.01); the high modification level threshold was 0.7 (--high-thresh 0.7); the minimum sites required for considering a motif were 50 (--min-sites 50); the lowest proportion of genomic sites with high modification level for motif detection was 0.7 (--min-frac-mod 0.7); the log-odds threshold for motif sequence enrichment was 1.6 (--min-log-odds 1.6); “--skip-search” was used to enable rare motif identification; the sequencing coverage threshold was 20× (--min-coverage 20). A paired *t*-test was used to compare the unique DNA methylation motif number between growth phases. For Snappy [20], the locus-based bedMethyl file generated by ONT Modkit v0.4.1 and the reference genome file were used for *de novo* DNA methylation motif discovery with the default settings.

### 4.10. DNA Methylation Proportion Within Motifs

After DNA methylation motif identification, two common motifs in *E. coli* (5′-G^m6^ATC-3′ and 5′-C^m5^CWGG-3′) and one motif in *L. acidophilus* (5′-GC^m6^ATC-3′) were selected for motif-specific DNA methylation level analysis. The NCBI RefSeq genome or assembled genome was used as a reference genome for DNA methylation calling. Using the BAM file and reference genome file as input, DNA methylation was called by specifying a motif in ONT Modkit v0.4.1 to generate a bedMethyl table, defined as a motif-based approach. Motif positions with coverage lower than 20× were removed.

All occurrences of the relevant motif in the bedMethyl table, including positions with or without detected modified bases, were utilized in the analysis. First, all occurrences of a selected motif were analyzed without separation by genome loci, defined as genome-level DNA methylation. The genome-level DNA methylation proportion of each motif was calculated by dividing the sum of methylated base numbers (N_mod_) by the sum of methylated and unmethylated base numbers (N_valid_cov_) across all motif-related sites. A paired *t*-test was used to compare the genome-level DNA methylation proportions between the stationary and exponential phases.

The analysis of each occurrence of identified motifs was then performed. Only motif positions present in both growth phases after coverage filtering were included in the comparison. The methylation proportion of each motif position was calculated by dividing the methylated base number (N_mod_) by the total number of both methylated and unmethylated bases (N_valid_cov_) of each position in the bedMethyl table. The methylation proportions of each motif position were used for principal component analysis (PCA).

### 4.11. DNA Methylation Proportion per Segment

The NCBI RefSeq genome or assembled genome was used as a reference genome for the analysis. The reference genome was separated into 10 kb segments using BEDTools v2.30.0 [58] and stored as a BED file. The locus-based bedMethyl tables were used for the analysis. All positions in the bedMethyl table, including positions with or without detected modified bases, were utilized. Genomic positions with coverage lower than 20× were removed. The methylation level of each segment was calculated by dividing the sum of methylated base numbers (N_mod_) by the sum of methylated and unmethylated base numbers (N_valid_cov_) within a 10 kb region. DNA methylation analysis was restricted to m6A and m5C in *E. coli* and m6A in *L. acidophilus*. Only segments present in both growth phases were included in the comparison. A paired *t*-test was used to compare the 10 kb segmental DNA methylation levels between the stationary and exponential phases. An unpaired *t*-test was performed to compare the methylation levels of 10 kb windows between different DNA methylation forms. *p*-value was adjusted by the Holm–Bonferroni method for multiple group comparisons.

### 4.12. DNA Methylation Proportion Around the DNA Replication Origin and Terminus

We examine the DNA methylation variance in regions around *ter* and *oriC* in two analyses: (1) methylation levels within the 100 kb flanking windows of *oriC* or *ter*; (2) the 10 kb segment methylation level changes across the genome. DNA methylation analysis was restricted to m6A and m5C in *E. coli* and m6A in *L. acidophilus*. Genomic positions with coverage lower than 20× were removed.

The DNA methylation levels of the 100 kb flanking regions and remaining genomic regions were calculated. A paired *t*-test was used to compare the methylation level of the 100 kb region flanking *oriC* or *ter* and the methylation level in the rest of the genome between the stationary and exponential phases. An unpaired *t*-test was performed to compare the methylation level of the 100 kb region flanking *oriC* or *ter*, as well as the remaining genomic regions, between different DNA methylation forms. *p*-value was adjusted by the Holm–Bonferroni method for multiple group comparisons.

The distance to *ter* or *oriC* was defined as the distance between the midpoint of a 10 kb genomic segment and *ter* or *oriC*. The differential DNA methylation level of each 10 kb window was calculated by the formula below:

$$M=\log_{2}\left((A+1)/(B+1)\right)$$

where *M* is the differential DNA methylation level of a 10 kb segment; *A* is the mean DNA methylation level from the stationary phase of a 10 kb segment; *B* is the mean DNA methylation level from the exponential phase of a 10 kb segment; two pseudo-counts of 1 are added to the formula to avoid zero values. The Spearman correlation coefficients between the distance to *ter* and the differential DNA methylation level were calculated.

To evaluate the effects of sequencing depth and growth phase on DNA methylation levels, a linear mixed model was constructed as follows:

m ~ a+b+c+(1 | d)

where *m* is the methylation level of each 10 kb segment; *a* is the growth phase; *b* is the scaled sequencing depth of each 10 kb segment; *c* is the modification form; and *d* is the replicate identifier. The model was performed with the “bobyqa” optimizer and evaluated using analysis of variance (ANOVA). The *p*-value threshold was 0.05.

### 4.13. Identification of Differentially Methylated Sites and Genes

ONT Modkit v0.4.1 was applied to the locus-based or motif-based bedMethyl table to identify the differentially methylated positions between growth phases, with a minimum coverage threshold of 20× for noise reduction. To allow comparable analysis, motif-based and locus-based differentially methylated site identification utilized an identical maximum coverage setting to calculate the estimated MAP-based *p*-value. Differential methylation analysis was restricted to m6A and m5C in *E. coli* and m6A in *L. acidophilus*. Using the genome size as a background, Fisher’s Exact Test was used to evaluate the overlap between locus-based and motif-based differentially methylated positions.

AGAT v1.4.1 [59] was used to convert the *E. coli* (GCF_000008865) and *L. acidophilus* (GCF_003047065.1) GFF annotation files to tsv files. A bash script filtered the tsv file for “source_tag” as “Refseq” and “primary_tag” as “gene”, followed by locating the gene promoter or the gene body regions considering strand orientation. For assembled genomes, the converted tsv files were filtered for “source_tag” as “AGAT” and “primary_tag” as “gene”. The differentially methylated positions outside of the gene body and promoter regions were also located. In this study, the promoter region was defined as the −35 to −7 frame upstream of the open reading frame start site, while the gene body referred to the region from the open reading frame start to the end site. The “balanced MAP-based *p*-value” of the bedMethyl file was used to identify the differentially methylated positions with a threshold of 0.05. The differentially modified positions (including chromosome, position, and strand) were aligned with gene promoter or gene body regions in the extracted annotation tsv file to identify differentially methylated genes. The differentially methylated sites were divided by the genomic GC content for normalization.

### 4.14. Identification of Differentially Expressed Genes

FastQC v0.12.1 [60] was used to perform quality control of the raw RNA sequencing data from *E. coli*, followed by adapter trimming and filtering using fastp v0.24.0 [61] with default settings. The filtered data were aligned to the *E. coli* RefSeq genome (GCF_000008865) or assembled genome by STAR v2.7.11b [62]. After feature read counting with HTSeq v2.0.5 [63], DESeq2 v1.44.0 [64] was used to perform the differentially expressed gene analysis with a Benjamini–Hochberg adjusted *p*-value threshold at 0.05.

### 4.15. Functional Annotation Analysis

The Entrez Gene IDs of differentially methylated or expressed genes were extracted from the NCBI RefSeq genome annotations and uploaded to the NCBI Database for Annotation, Visualization, and Integrated Discovery (DAVID) [65] for functional annotation analysis. For *E. coli*, the analysis was performed using Gene Ontology and KEGG databases with *E. coli* O157:H7 str. Sakai as a background. Classification stringency was selected as “Medium” for functional annotation clustering with the modified Fisher Exact *p*-value threshold at 0.05, the minimum enrichment score at 2.00, and the initial and final group memberships at 2. For *L. acidophilus*, the functional annotation analysis was performed using the Gene Ontology database under the *L. acidophilus* background, with the modified Fisher Exact *p*-value threshold at 0.1.

### 4.16. Association Between DNA Methylation and Gene Expression

A customized R script was used to identify the differentially methylated and expressed genes in *E. coli* based on the RefSeq genome annotations (GCF_000008865). Using the gene ID number in the annotation tsv file as a background, Fisher’s Exact Test was performed to evaluate if the overlap between the differentially methylated gene set and the differentially expressed gene set was more than expected by chance.

We then examined the relationship between methylation and gene expression in the set of genes that were both differentially methylated and differentially expressed. Specifically, we looked at whether the methylation status of a gene (gene body and promoter) significantly affected the gene expression level. Accordingly, a linear model including the replicate and growth phase effects was applied separately to each gene that was both differentially methylated and differentially expressed in the *E. coli* dataset. Only the differentially methylated genes identified by the locus-based approach were included. The model was performed on each gene using the formula below:

$$y=\beta_{1}X_{1}+\beta_{2}X_{2}+\beta_{3}X_{3}$$

where *y* is the expression vector of a gene *n* (*individual*) × 1; *β*_1_ is the regression coefficient for evaluating the association between DNA methylation and gene expression; *X*_1_ is the DNA methylation proportion matrix *n* (*individual*) × *m* (*differentially methylated position*) of a gene; *X*_2_ is the growth phase vector *n* (*phase*) × 1; *X*_3_ is the replicate vector *n* (*replicate*) × 1. The coverage threshold for $X_{1}$ matrix was 0×. The *p*-value (denoted as *P_T_*) between the methylation level and the expression level of the related gene was calculated from ANOVA by comparing the models with and without *X*_1_. Considering the small number of differentially methylated sites for each gene, bootstrapping was used to correct the *p*-value, because it accounts for non-normal distributions in the data. The model was bootstrapped 1000 times by randomly sampling the gene expression vector, and then calculating the significance of the methylation status effect on the randomly shuffled expression level. The array of randomly bootstrapped *p*-values was used to create a distribution of expected *p*-values where there was no true relationship, denoted as *P_R_*. The *P_T_* values equal to or below the one-tailed 95% confidence interval of the *P_R_* distribution were considered significant.

Genes with differentially methylated positions showing a strong linear correlation with gene expression were identified based on this significance. The Entrez Gene IDs of these genes were uploaded to the NCBI DAVID [65] for functional annotation analysis using the Gene Ontology and KEGG databases under the *E. coli* O157:H7 str. Sakai background, with classification stringency as “Medium” and the modified Fisher Exact *p*-value threshold at 0.05.

## 5. Conclusions

The genome-wide DNA fragment density and DNA methylation shifts in *E. coli* and *L. acidophilus* during the growth phase transition were examined by Oxford Nanopore sequencing in this study. DNA methylation levels (N6-methyladenine and 5-methylcytosine) were globally and regionally lower during the exponential phase compared to the stationary phase. In *E. coli*, differentially methylated genes can robustly estimate the overall transcriptomic profile. The inclusion of multiple DNA methylation forms and all methylated loci can improve concordance with differentially expressed genes. Our findings reveal that the bacterial DNA methylation pattern shifts during the growth phase transition, and indicate that methylation could be an accessible complementary method for gene expression estimation.

## Figures and Tables

**Table 1:** Bacterial data of this study.

Bacteria	Dataset	Phase (Collection Hour)	Replicates	DNA N50 (Kbp) ^3^	DNA Coverage (x) ^3^	RNA Reads (Million) ^4^
*Escherichia coli* ^1^	Original	exponential (3)	5	48.02 ± 5.28	248.60 ± 217.32	165.28 ± 12.40
		stationary (15)	5	50.58 ± 7.32	497.20 ± 711.53	192.70 ± 21.26
	Validation	exponential (3)	3	48.29 ± 0.07	354.59 ± 57.00	NA
		stationary (15)	3	49.50 ± 0.50	384.50 ± 134.91	NA
*Lactobacillus acidophilus* DSM 20079 ^2^	Original	exponential (20.5)	3	0.42 ± 0.07	740.33 ± 119.08	NA
		stationary (48)	3	2.28 ± 0.55	304.00 ± 126.57	NA

^1^ Chicken fecal samples. ^2^ Commercial strain from humans. ^3^ Data from the primary mapped DNA reads showed as mean ± standard deviation. ^4^ Data from the raw RNA reads of replicate 1, 2, and 3 from the original dataset only.

## Data Availability

The raw sequence data of this study were archived under the NCBI BioProject: PRJNA1268599. Sample accession numbers are listed in Table S40. Summary and statistics tables are available in Tables S1–S40, and additional supporting analyses results are shown in Figures S1–S26. Coding scripts for this study were available on the GitHub repository: https://github.com/SimonChen1997/DNA_methylation_starvation_response (accessed on 9 September 2026). POD5 data for this study were available on the UQ eSpace repository: https://doi.org/10.48610/7bdba4e.

## Supplementary Materials

The following supporting information can be downloaded at: https://www.mdpi.com/article/10.3390/epigenomes10030057/s1.
