# Supplementary material for: Growth Phase-Related DNA Methylation and Localized DNA Fragment Density Variation in Escherichia coli and Lactobacillus acidophilus Genomes
Source: Epigenomes. 2026 Sep 11;10(3):57. doi: 10.3390/epigenomes10030057 (PMC13605387; doi:10.3390/epigenomes10030057)
Supplement: Supplementary file 1 [file epigenomes-10-00057-s001.zip › Supplemental_Files/Supplemental_Figures.pdf]

Growth phase-related DNA methylation and localized DNA fragment density variation in *Escherichia coli* and *Lactobacillus acidophilus* genomes

Supplemental Figures

Table of Contents

Figure S1 1

Figure S2 2

Figure S3 3

Figure S4 5

Figure S5 6

Figure S6 7

Figure S7 8

Figure S8 9

Figure S9 11

Figure S10 13

Figure S11 14

Figure S12 15

Figure S13 16

Figure S14 17

Figure S15 18

Figure S16 19

Figure S17 20

Figure S18 21

Figure S19 23

Figure S20 24

Figure S21 25

Figure S22 26

Figure S23 27

Figure S24..28

Figure S25..29

Figure S26..30

Figure S1

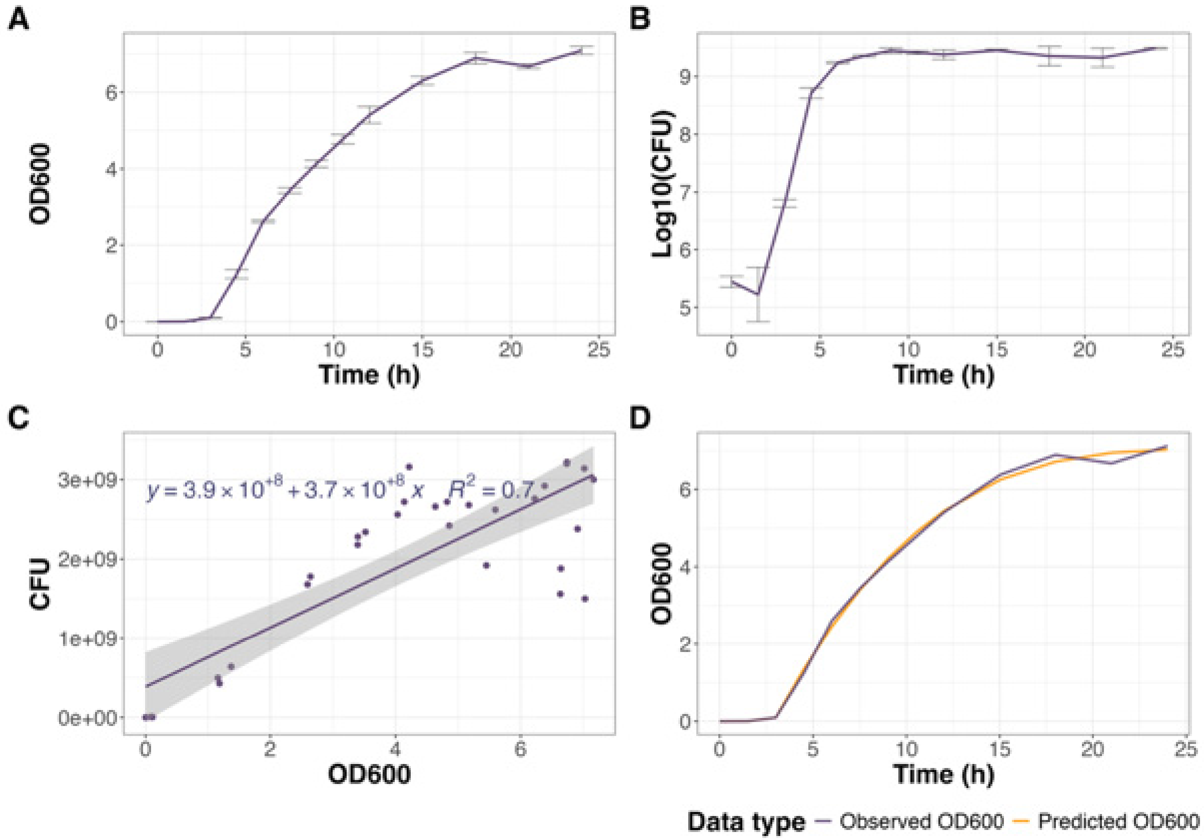

Figure S1. Growth curve analyses of *Escherichia coli*. The observed growth curves at (A) OD600 and (B) colony-forming unit values. (C) The correlation between observed OD600 and colony-forming unit values, and (D) the observed and predicted growth curves. Analysis here only included data in *E. coli*. The *E. coli* suspension was collected every 1.5 hours during the first 12 hours and 3 hours over the subsequent 12 hours. A linear regression analysis was performed to evaluate the association between OD600 and colony-forming unit values. The OD600 values at 0.5-hour intervals were imputed using Gompertz statistical models, with the observed OD600 values as input.

Figure S2

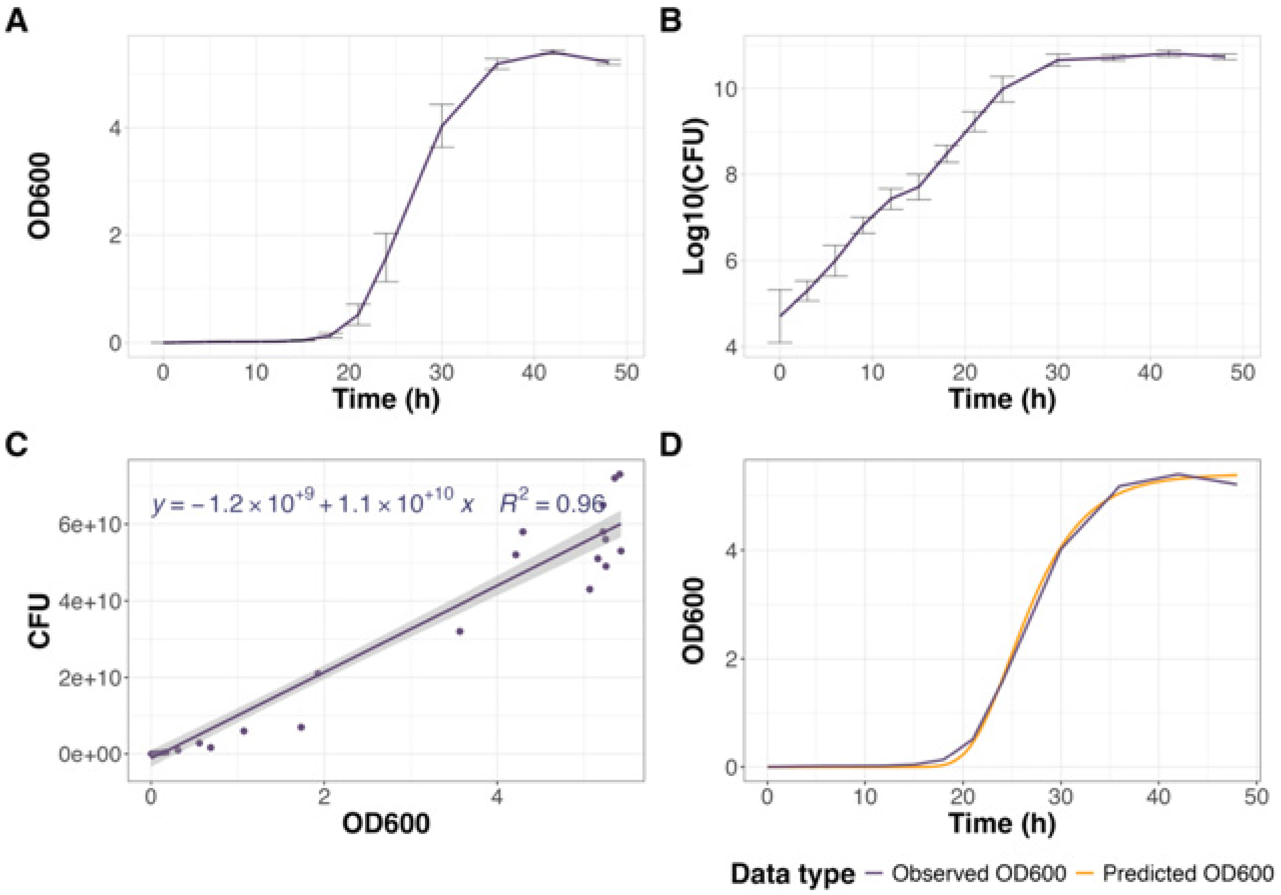

Figure S2. Growth curve analyses of *Lactobacillus acidophilus*. The observed growth curves at (A) OD600 and (B) colony-forming unit values. (C) The correlation between observed OD600 and colony-forming unit values, and (D) the observed and predicted growth curves. Analysis here only included data in *L. acidophilus*. The *L. acidophilus* suspension was collected every 3 hours during the first 24 hours and 6 hours over the next 24 hours. A linear regression analysis was performed to evaluate the association between OD600 and colony-forming unit values. The OD600 values at 0.5-hour intervals were imputed using Gompertz statistical models, with the observed OD600 values as input.

Figure S3

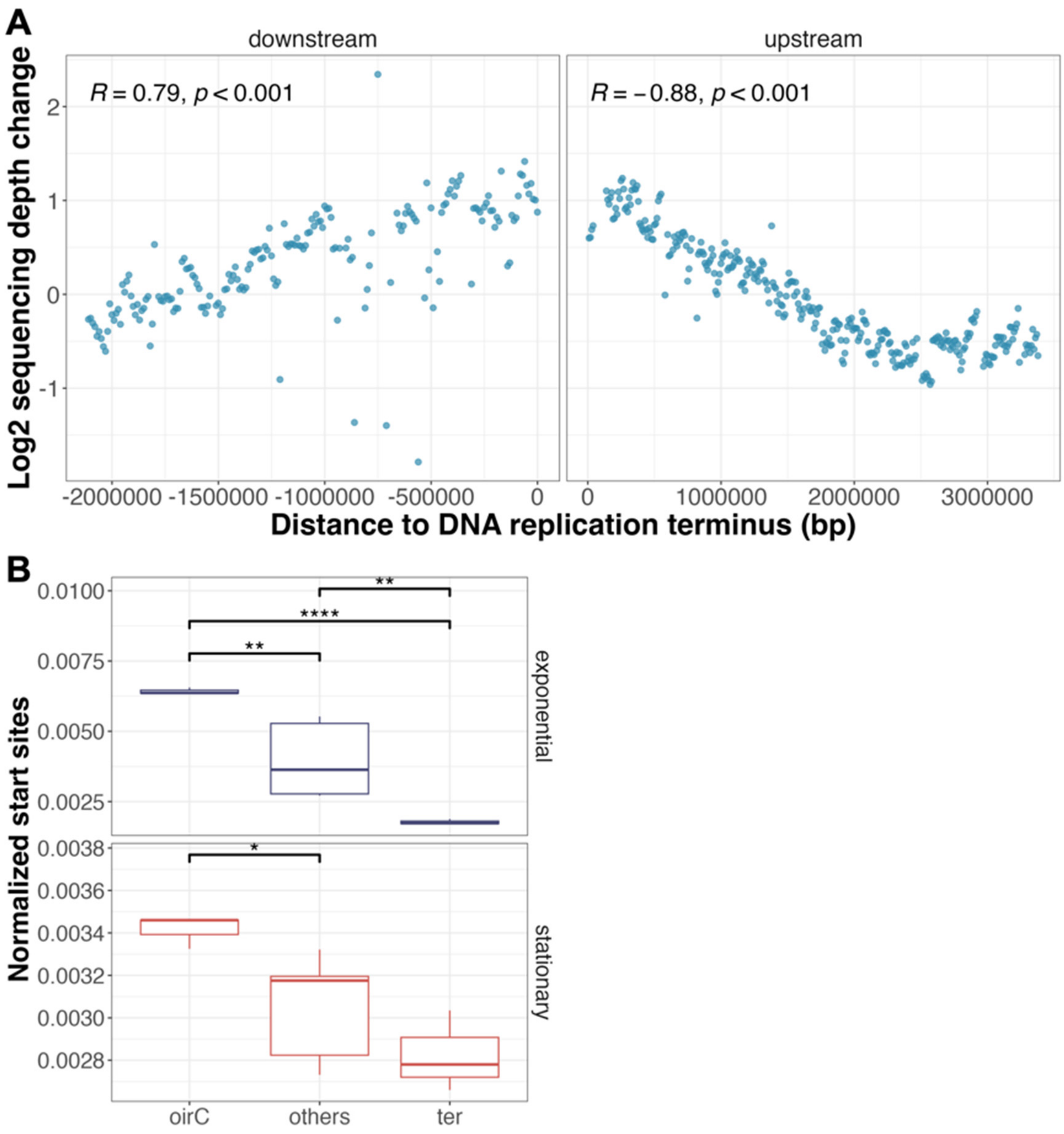

Figure S3. Regional DNA fragment density analyses of *Escherichia coli*. (A) Sequencing depth changes of each 10-kb window between the exponential and stationary phases around the DNA replication terminus. (B) The start site difference between the exponential and stationary phases. Analysis here only included replicates 1, 2, and 3 with DNA and RNA sequencing data in *E. coli*. The RefSeq genome (GCF_000008865) was used for the analysis. Only the data from the chromosome NC_002695.2 were included. The log2 value change is the base-2 logarithm of the stationary value relative to the exponential value. The Spearman correlation coefficient was calculated to estimate the relation between distance to the DNA replication terminus and log2 sequencing depth change. The start sites were normalized by the genome GC

content. An unpaired t-test was used to compare the normalized start sites among different regions. *ter*: DNA replication terminus 100-kb flanking region. *oriC*: DNA replication origin100-kb flanking region. Others: regions outside the DNA replication origin and terminus 100-kb flanking regions. *P-values* < 0.05: *; *P-values* < 0.01: **; *P-values* < 0.001: ***.

Figure S4

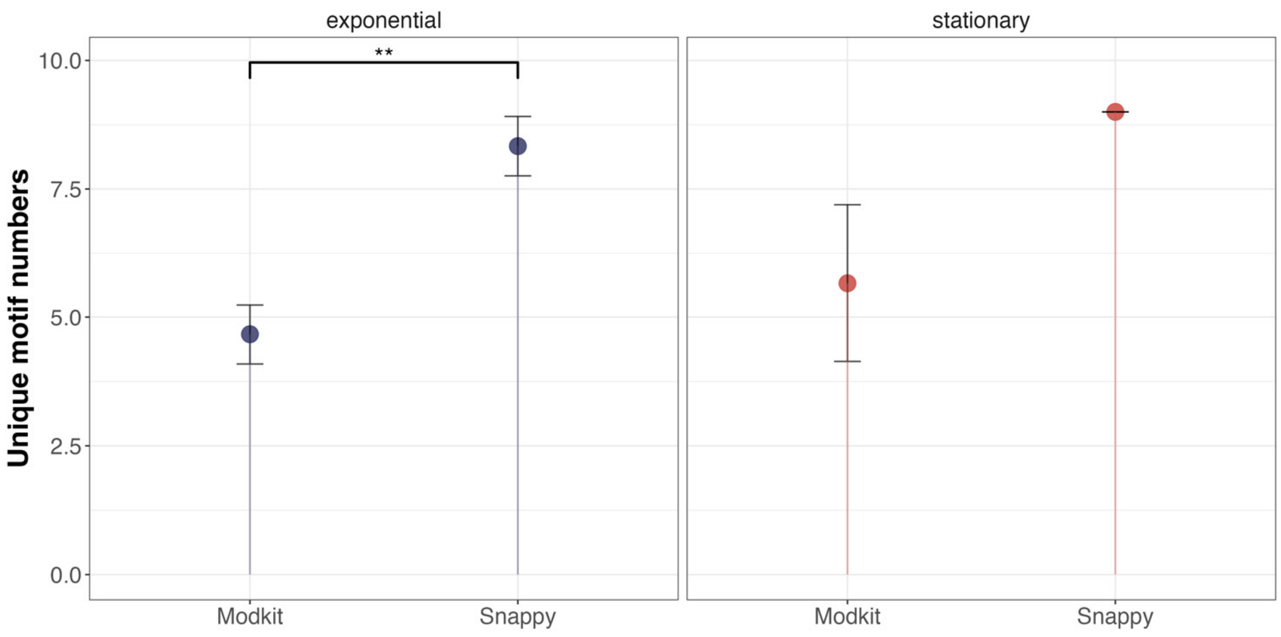

Figure S4. The unique DNA methylation motif number of two growth phases in *Escherichia coli* identified by Modkit and Snappy. Analysis here only included replicates 1, 2, and 3 with DNA and RNA sequencing data in *E. coli*. The RefSeq genome (GCF_000008865) was used for the analysis. The unique motif numbers are shown in the graph as mean ± standard deviation. *P-values* < 0.05: *; *P-values* < 0.01: **; *P-values* < 0.001: ***.

Figure S5

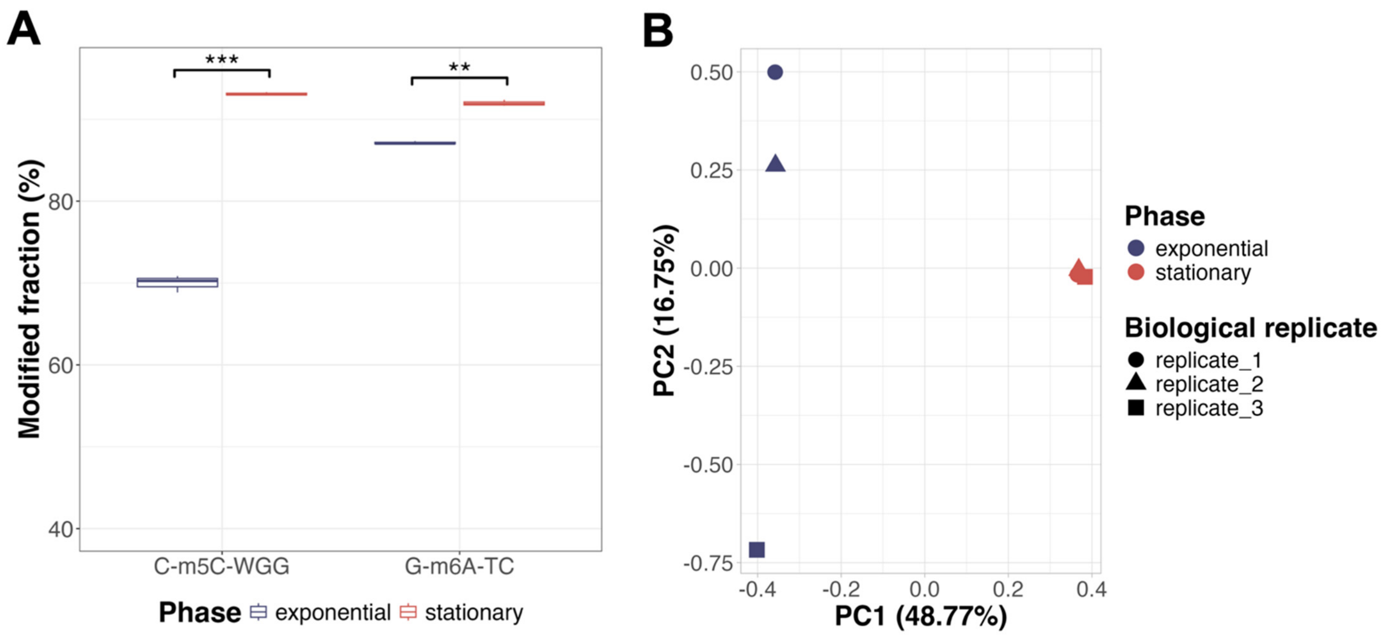

Figure S5. DNA methylation level analyses of *Escherichia coli* using the motif-based approach. (A) Genome-level DNA methylation proportions between the exponential and stationary phases. (B) Principal component analysis on base-level DNA methylation proportions of all selected motifs. Analysis here only included replicates 1, 2, and 3 with DNA and RNA sequencing data in *E. coli*. The RefSeq genome (GCF_000008865) was used for the analysis. A paired t-test was used to compare the modified fraction between different phases. *P-values* < 0.05: *; *P-values* < 0.01: **; *P-values* < 0.001: ***.

Figure S6

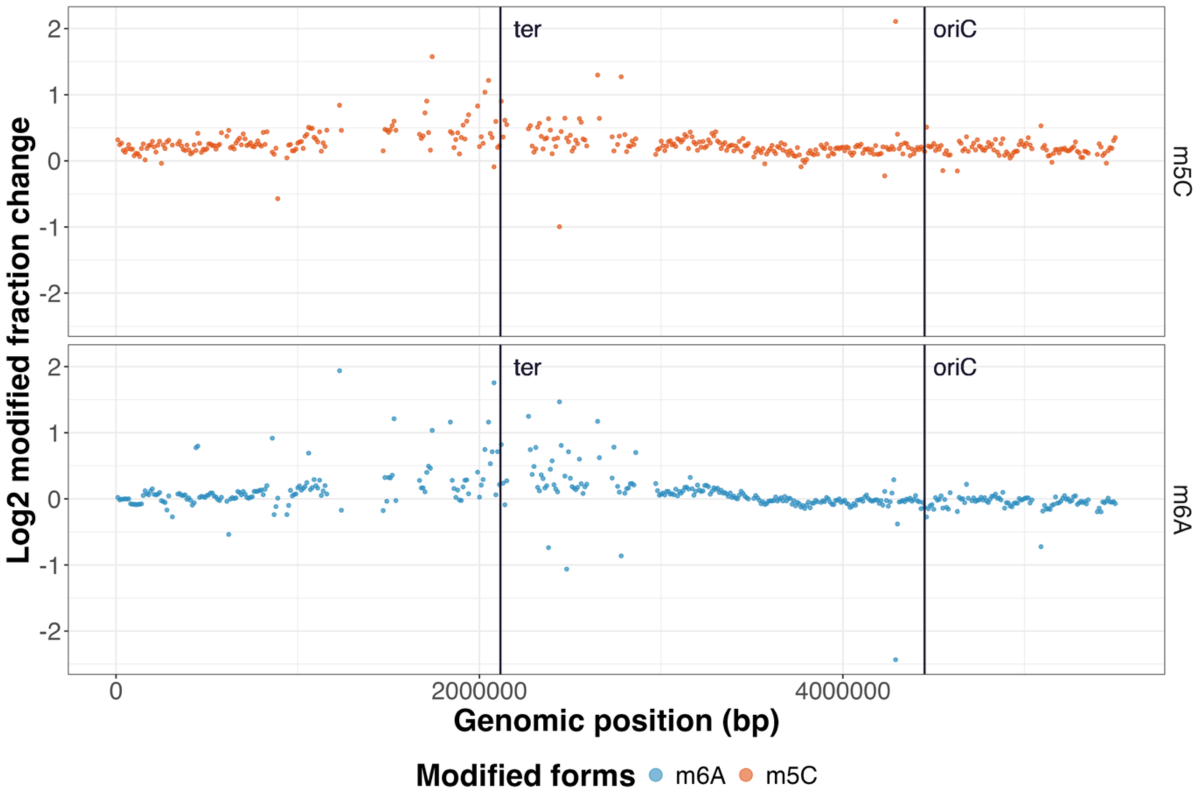

Figure S6. **DNA methylation level changes of each 10-kb window across the genome of *Escherichia coli* using the locus-based approach**. Analysis here only included replicates 1, 2, and 3 with DNA and RNA sequencing data in *E. coli*. The RefSeq genome (GCF_000008865) was used for the analysis. Only the data from the chromosome NC_002695.2 were included. The log2 value change is the base-2 logarithm of the stationary value relative to the exponential value. *ter*: DNA replication terminus. *oriC*: DNA replication origin. m6A: N6-methyladenine. m5C: 5-methylcytosine.

Figure S7

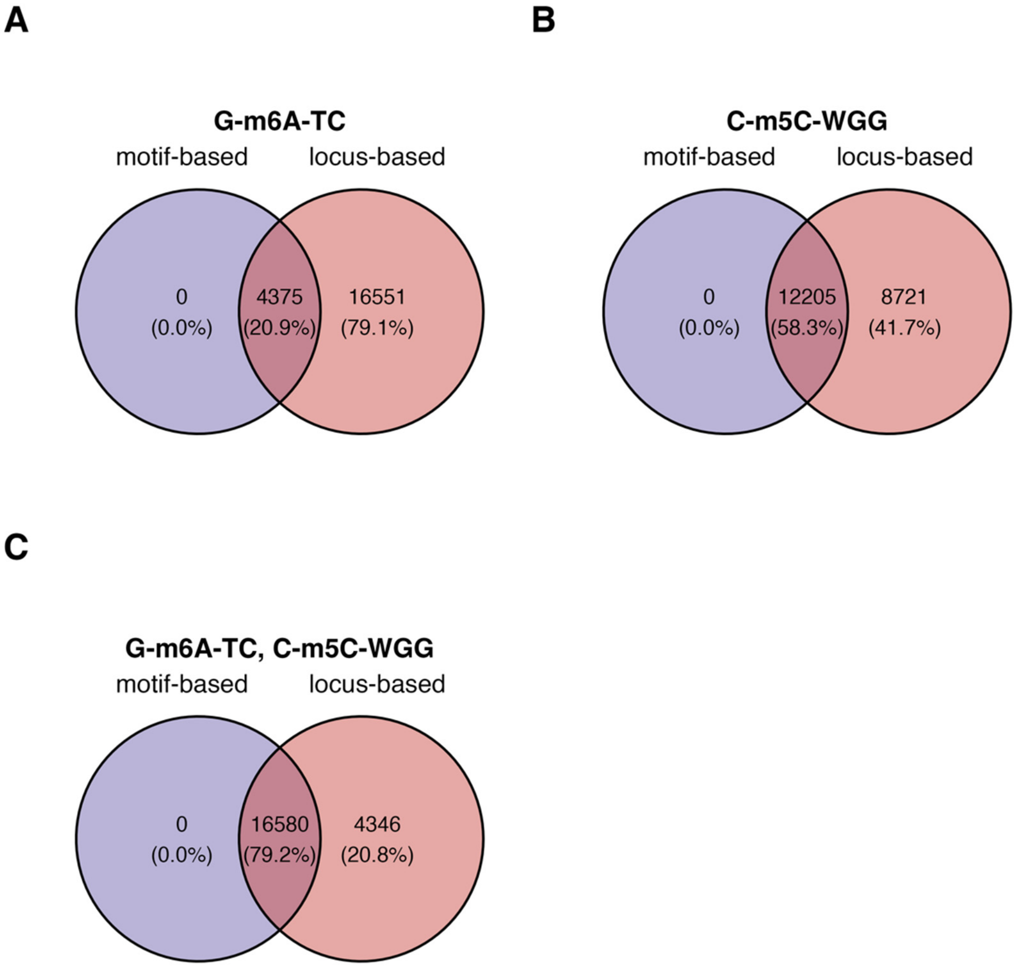

Figure S7. The shared differentially methylated positions of *Escherichia coli* between motif-based and locus-based approaches. Analysis here only included replicates 1, 2, and 3 with DNA and RNA sequencing data in *E. coli*. The RefSeq genome (GCF_000008865) was used for the analysis. The title in each graph indicated the motifs included in the motif-based approach. A Fisher's Exact Test was performed to evaluate the identified position overlap significance between the motif-based and locus-based approaches. m6A: N6-methyladenine. m5C: 5-methylcytosine.

Figure S8

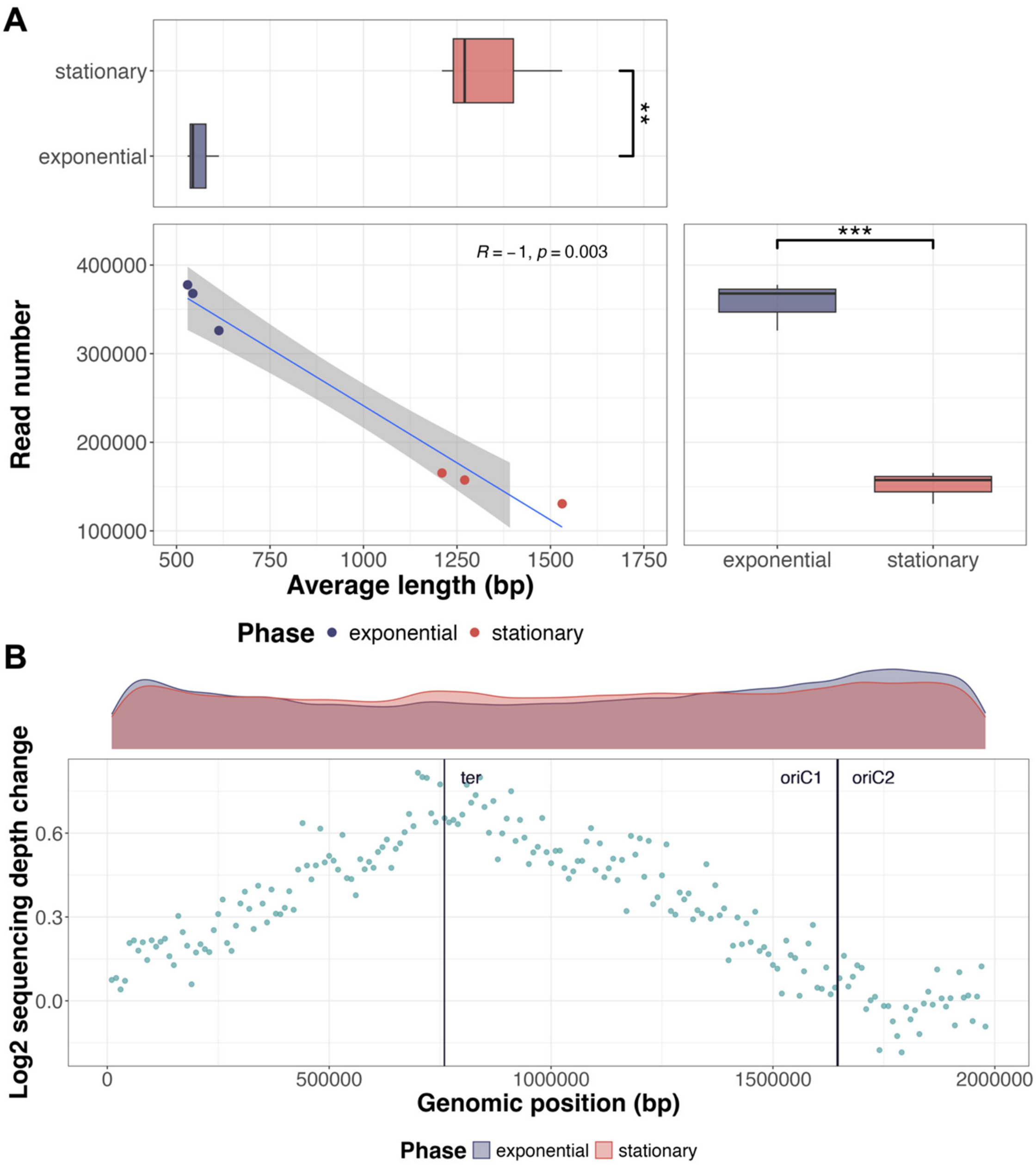

Figure S8. DNA fragment density analyses of *Lactobacillus acidophilus*. (A) Average DNA length and read number between the exponential and stationary phases. (B) Sequencing depth changes of each 10-kb window across the genome between the exponential and stationary phases. The side plot on the top of graph B is the DNA sequence start site density across the RefSeq genome (GCF_003047065.1). Only the data from the chromosome NZ_CP139575.1 were included in graph B. The log2 value change is the base-2 logarithm of the stationary value relative to the exponential value. The Spearman correlation coefficient was calculated to estimate the correlation between average DNA length and read number. A paired t-test was used

to compare the average length and read number between different phases. *ter*: DNA replication terminus. *oriC*: DNA replication origin. *P-values* < 0.05: *; *P-values* < 0.01: **; *P-values* < 0.001: ***.

Figure S9

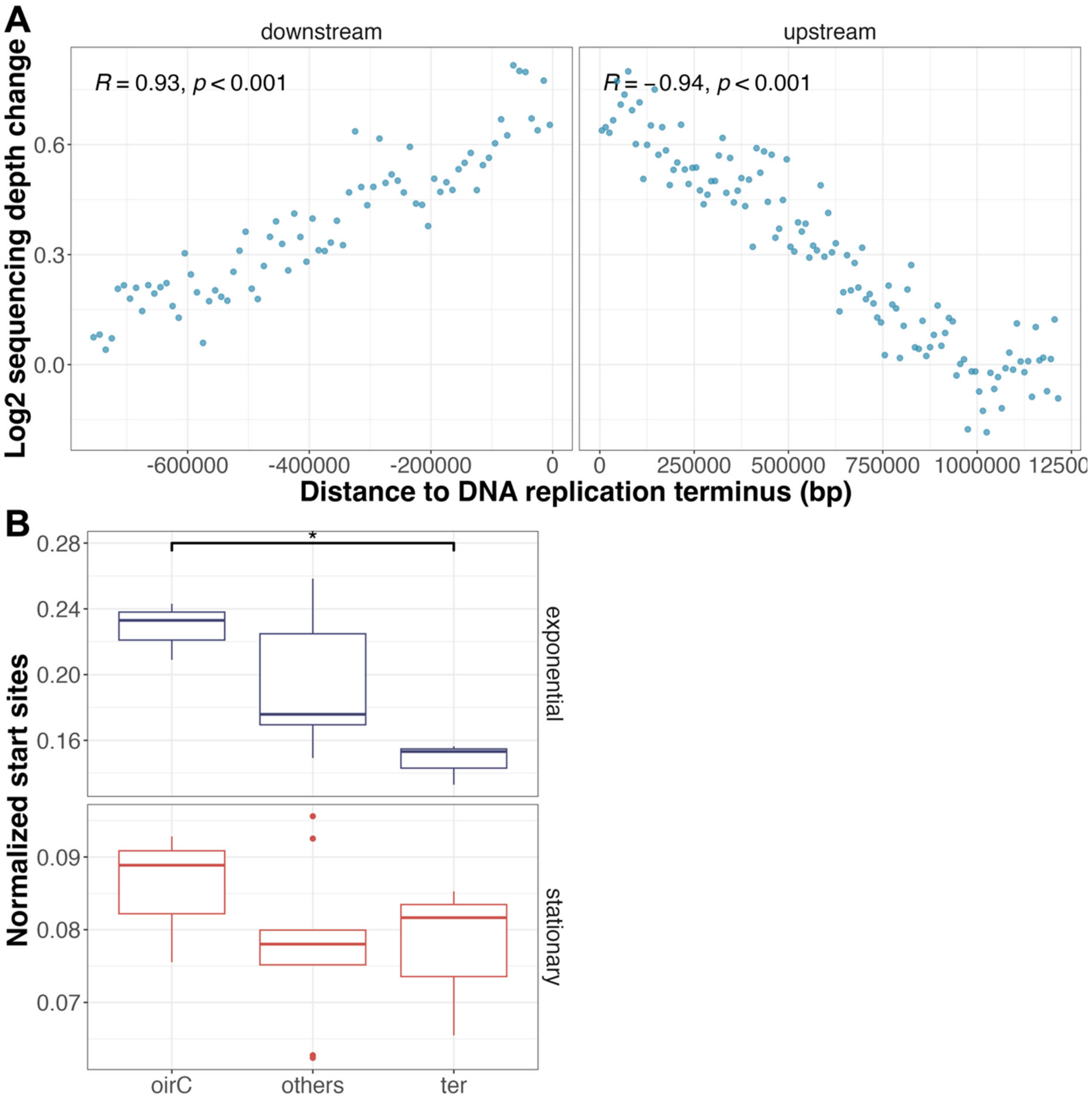

Figure S9. Regional DNA fragment density analyses of *Lactobacillus acidophilus*. (A) Sequencing depth changes of each 10-kb window between the exponential and stationary phases around the DNA replication terminus. **(B) The start site difference between the exponential and stationary phases**. The RefSeq genome (GCF_003047065.1) was used for the analysis. Only the data from the chromosome NZ_CP139575.1 were included. The log2 value change is the base-2 logarithm of the stationary value relative to the exponential value. The Spearman correlation coefficient was calculated to estimate the relation between distance to the DNA replication terminus and log2 sequencing depth change. The start sites were normalized

by the genome GC content. An unpaired t-test was used to compare the normalized start sites among different regions. *ter*: DNA replication terminus 100-kb flanking region. *oriC*: DNA replication origin 100-kb flanking region. Others: regions outside the DNA replication origin and terminus 100-kb flanking regions. *P-values* < 0.05: *; *P-values* < 0.01: **; *P-values* < 0.001: ***.

Figure S10

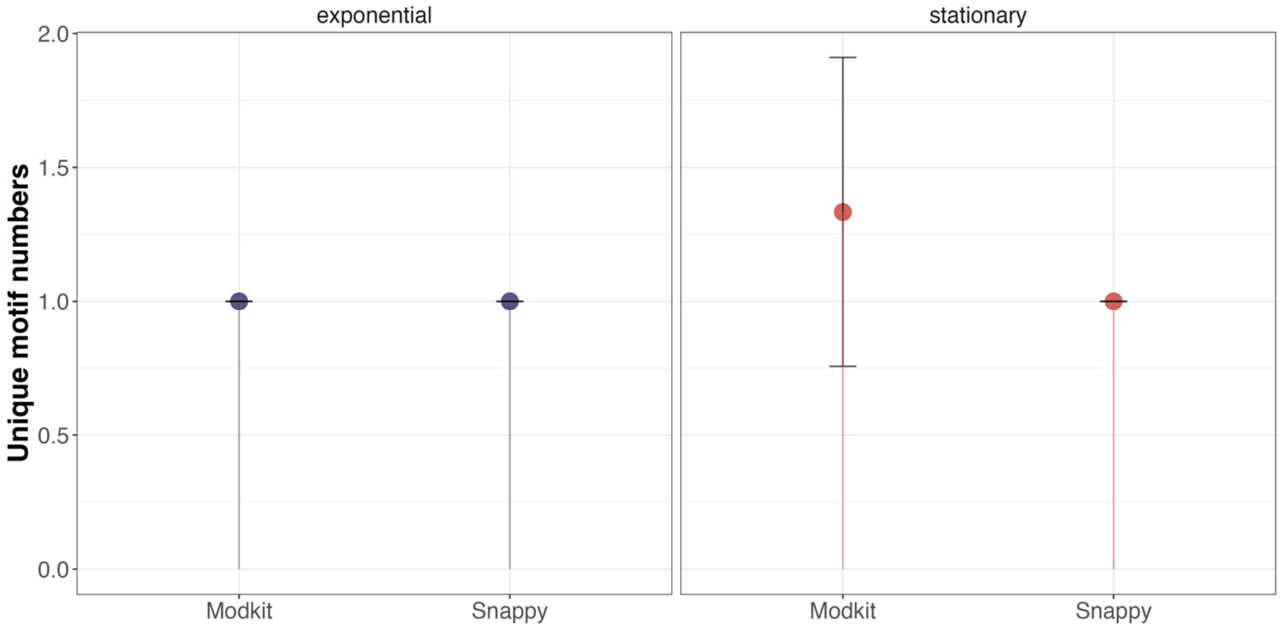

Figure S10. The unique DNA methylation motif number of two growth phases in *Lactobacillus acidophilus* identified by Modkit and Snappy. The RefSeq genome (GCF_003047065.1) was used for the analysis. The unique motif numbers are shown in the graph as mean ± standard deviation.

Figure S11

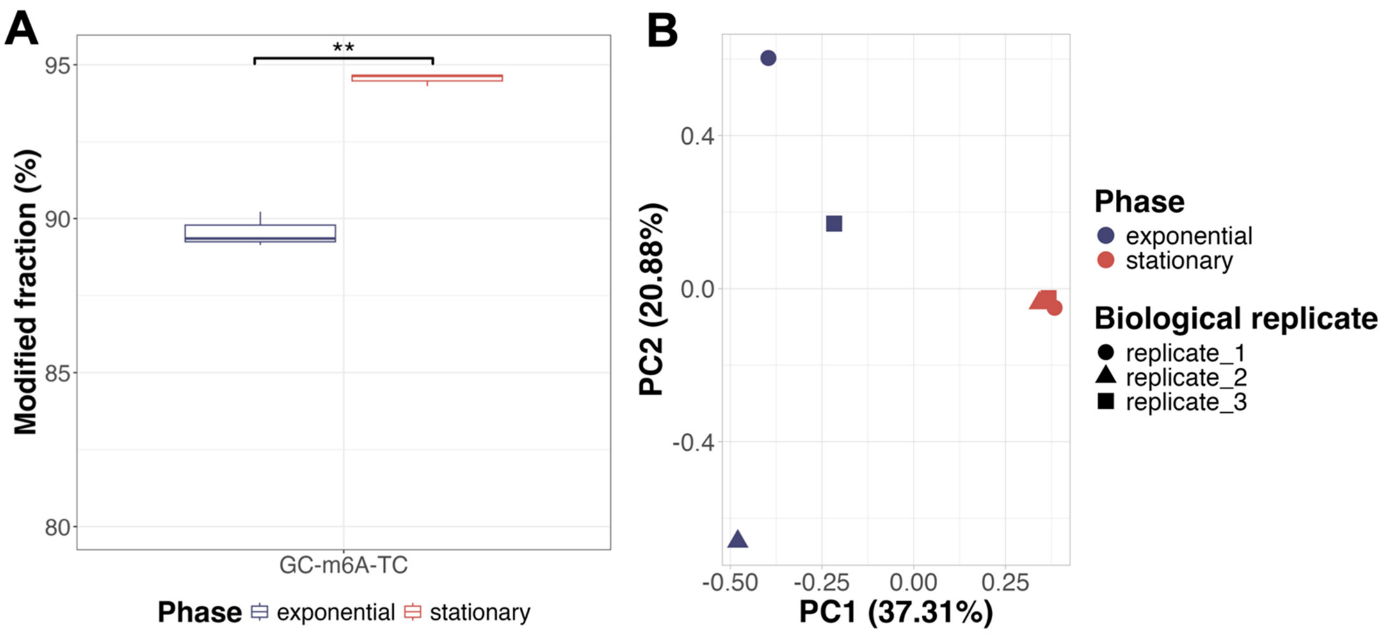

Figure S11. DNA methylation level analyses of *Lactobacillus acidophilus* using the motif-based approach. (A) Genome-level DNA methylation proportions between the exponential and stationary phases. (B) Principal component analysis on base-level DNA methylation proportions of all selected motifs. The RefSeq genome (GCF_003047065.1) was used for the analysis. A paired t-test was used to compare the modified fraction between different phases. *P-values* < 0.05: *; *P-values* < 0.01: **; *P-values* < 0.001: ***.

Figure S12

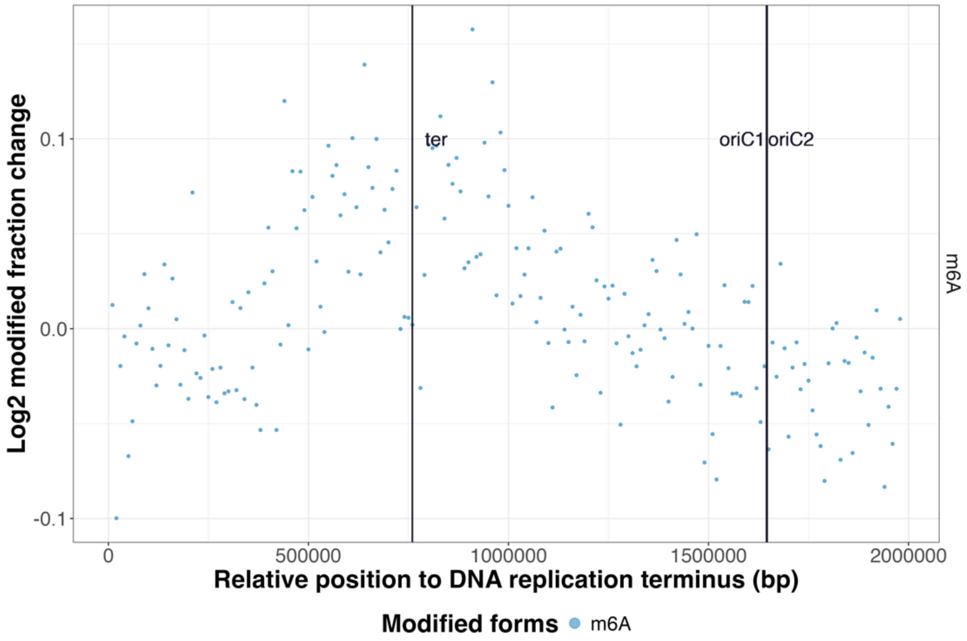

Figure S12. DNA methylation level changes of each 10-kb window across the genome of *Lactobacillus acidophilus* using the locus-based approach. The RefSeq genome (GCF_003047065.1) was used for the analysis. Only the data from the chromosome NZ_CP139575.1 were included. The log2 modified fraction change is the base-2 logarithm of the stationary value relative to the exponential value. *ter*: DNA replication terminus. *oriC*: DNA replication origin. m6A: N6-methyladenine.

Figure S13

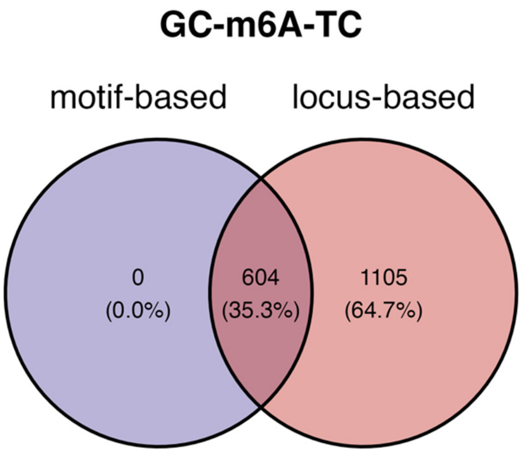

Figure S13. The shared differentially methylated positions of *Lactobacillus acidophilus* between motif-based and locus-based approaches. The title in the graph indicates the motifs included in the motif-based approach. The RefSeq genome (GCF_003047065.1) was used for the analysis. A Fisher's Exact Test was performed to evaluate the identified position overlap significance between the motif-based and locus-based approaches. m6A: N6-methyladenine.

Figure S14

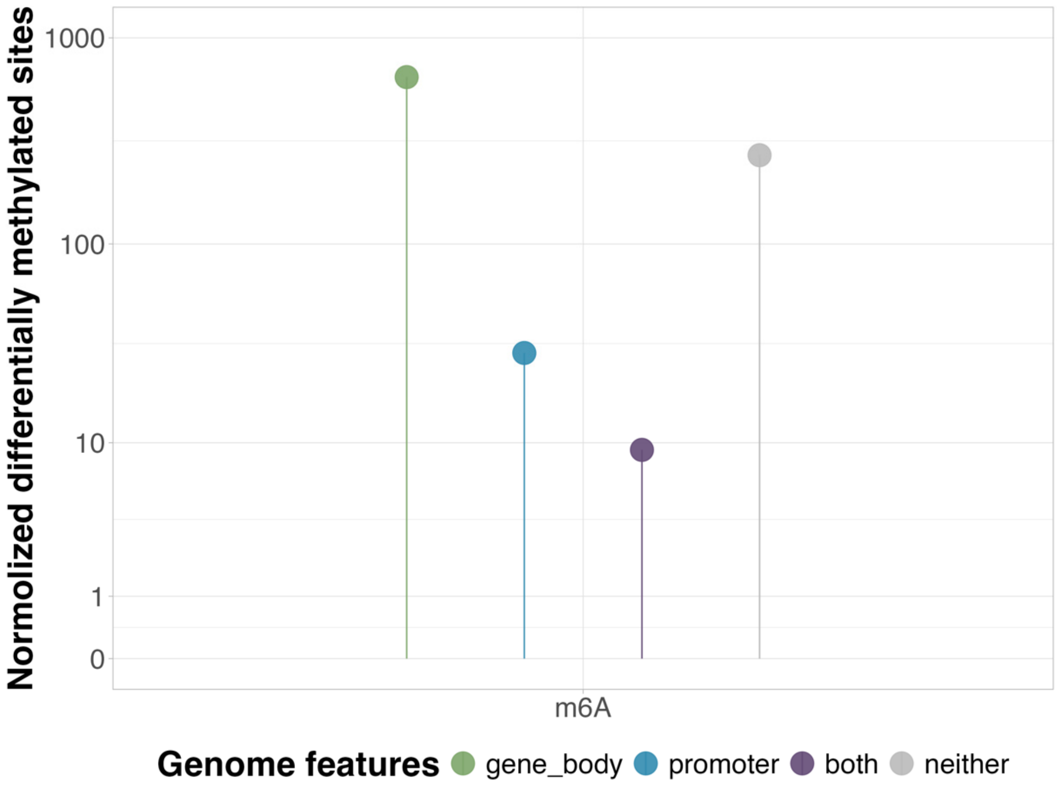

Figure S14. Differentially methylated position distribution of *Lactobacillus acidophilus* using the locus-based approach. The RefSeq genome (GCF_003047065.1) was used for the analysis. The differentially methylated sites were normalized by the background GC content. The promoter region was defined as the -35 to -7 frame upstream of the open reading frame start site, while the gene body referred to the region from the open reading frame start to the end site. m6A: N6-methyladenine.

Figure S15

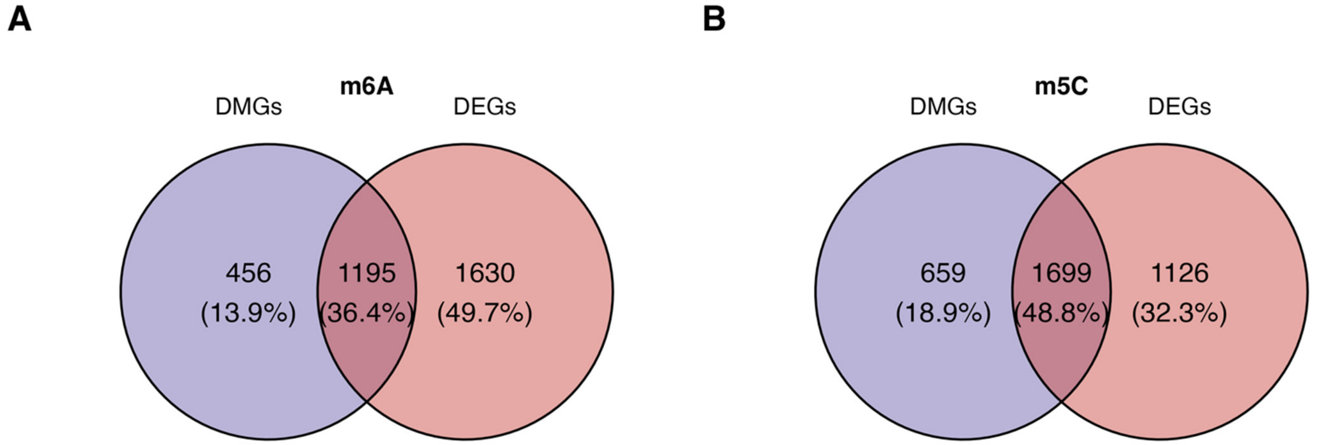

Figure S15. The shared gene number between the locus-based DNA methylation and transcriptomic profiles of *Escherichia coli* in (A) N6-methyladenine and (B) 5-methylcytosine. Analysis here only included replicates 1, 2, and 3 with DNA and RNA sequencing data in *E. coli*. The RefSeq genome (GCF_000008865) was used for the analysis. A Fisher's Exact Test was performed to evaluate the gene overlap significance between the DNA and RNA datasets. m6A: N6-methyladenine. m5C: 5-methylcytosine.

Figure S16

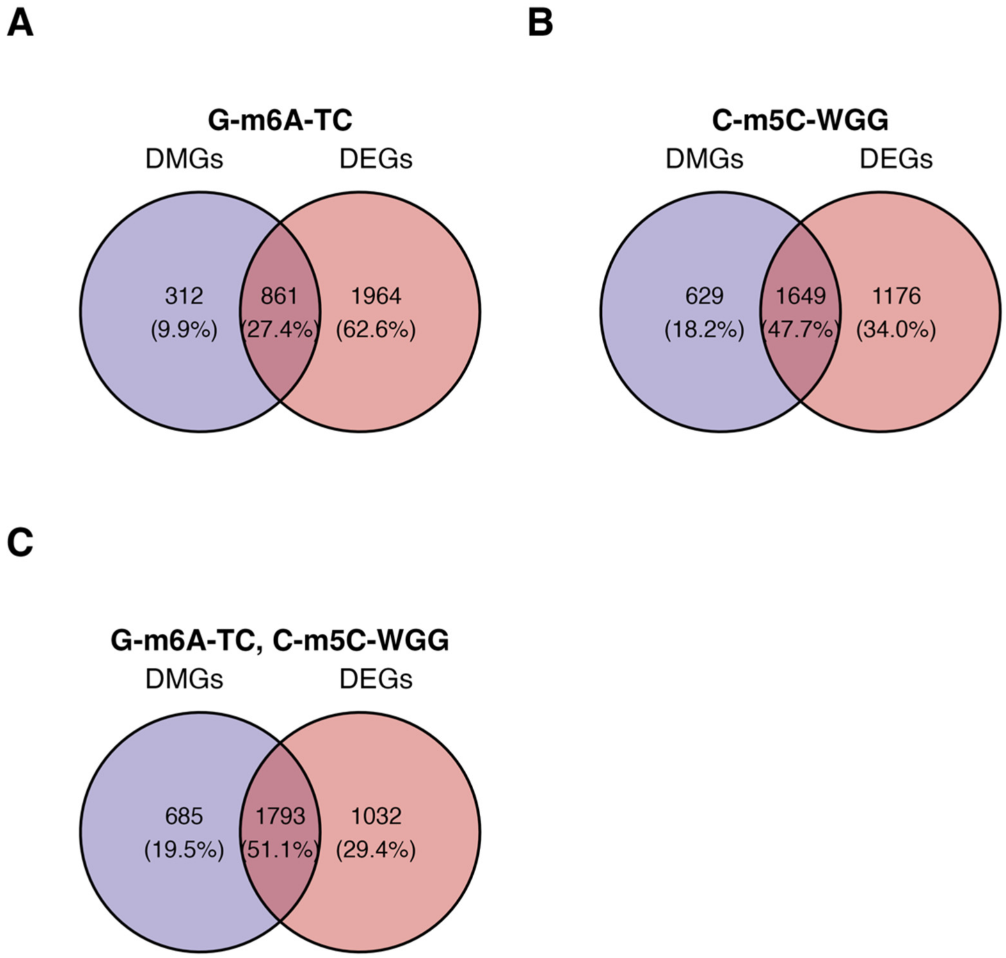

Figure S16. The shared gene number between the transcriptomic and motif-based DNA methylation profiles of *Escherichia coli*. Analysis here only included replicates 1, 2, and 3 with DNA and RNA sequencing data in *E. coli*. The RefSeq genome (GCF_000008865) was used for the analysis. The title in each graph indicated the motifs included in the motif-based approach. A Fisher's Exact Test was performed to evaluate the gene overlap significance between the DNA and RNA datasets. m6A: N6-methyladenine. m5C: 5-methylcytosine.

Figure S17

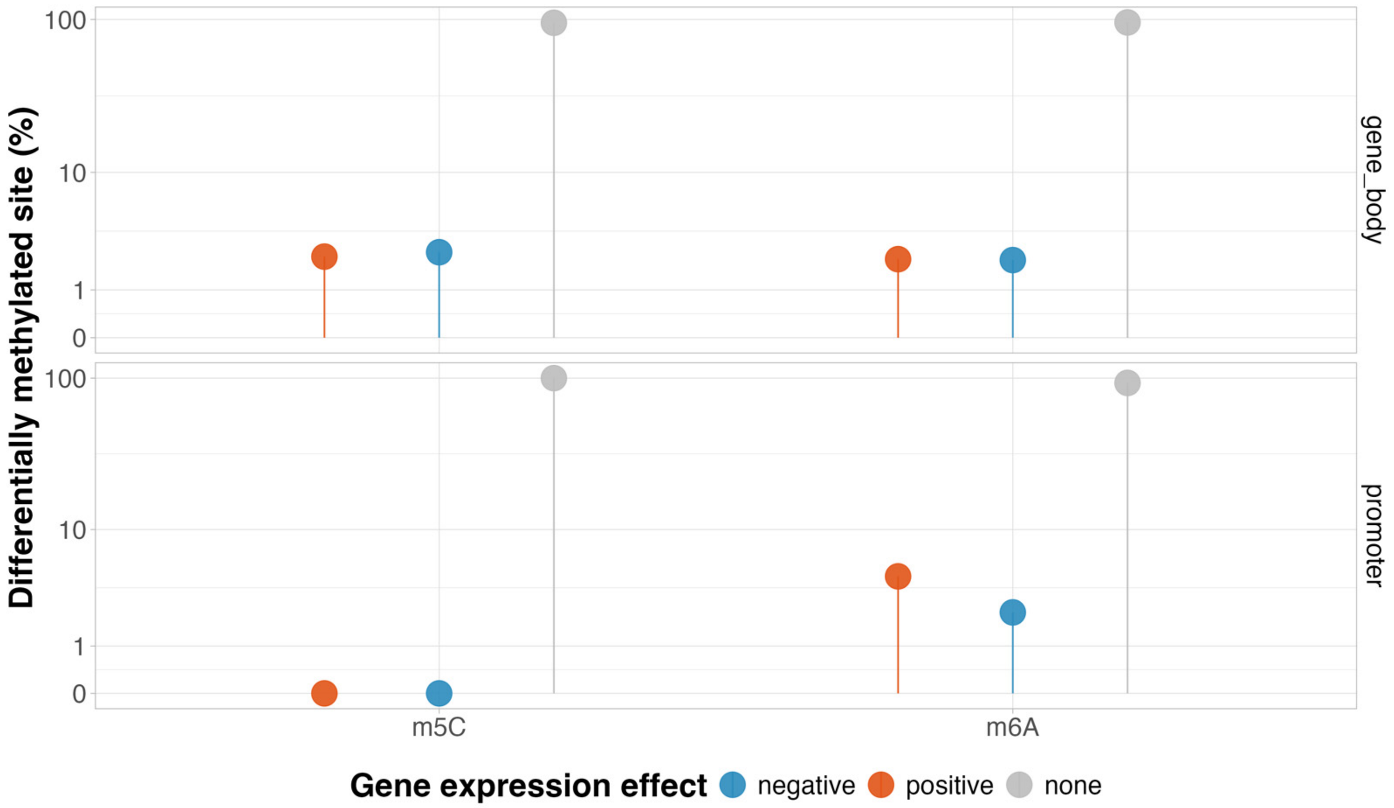

Figure S17. The proportions of differentially methylated genomic sites by gene expression effects in *Escherichia coli*. Analysis here only included replicates 1, 2, and 3 with DNA and RNA sequencing data in *E. coli*. The RefSeq genome (GCF_000008865) was used for the analysis. Results were derived from the locus-based approach. The coverage threshold for the DNA methylation matrix was 20x. The promoter region was defined as the -35 to -7 frame upstream of the open reading frame start site, while the gene body referred to the region from the open reading frame start to the end site. m6A: N6-methyladenine. m5C: 5-methylcytosine.

Figure S18

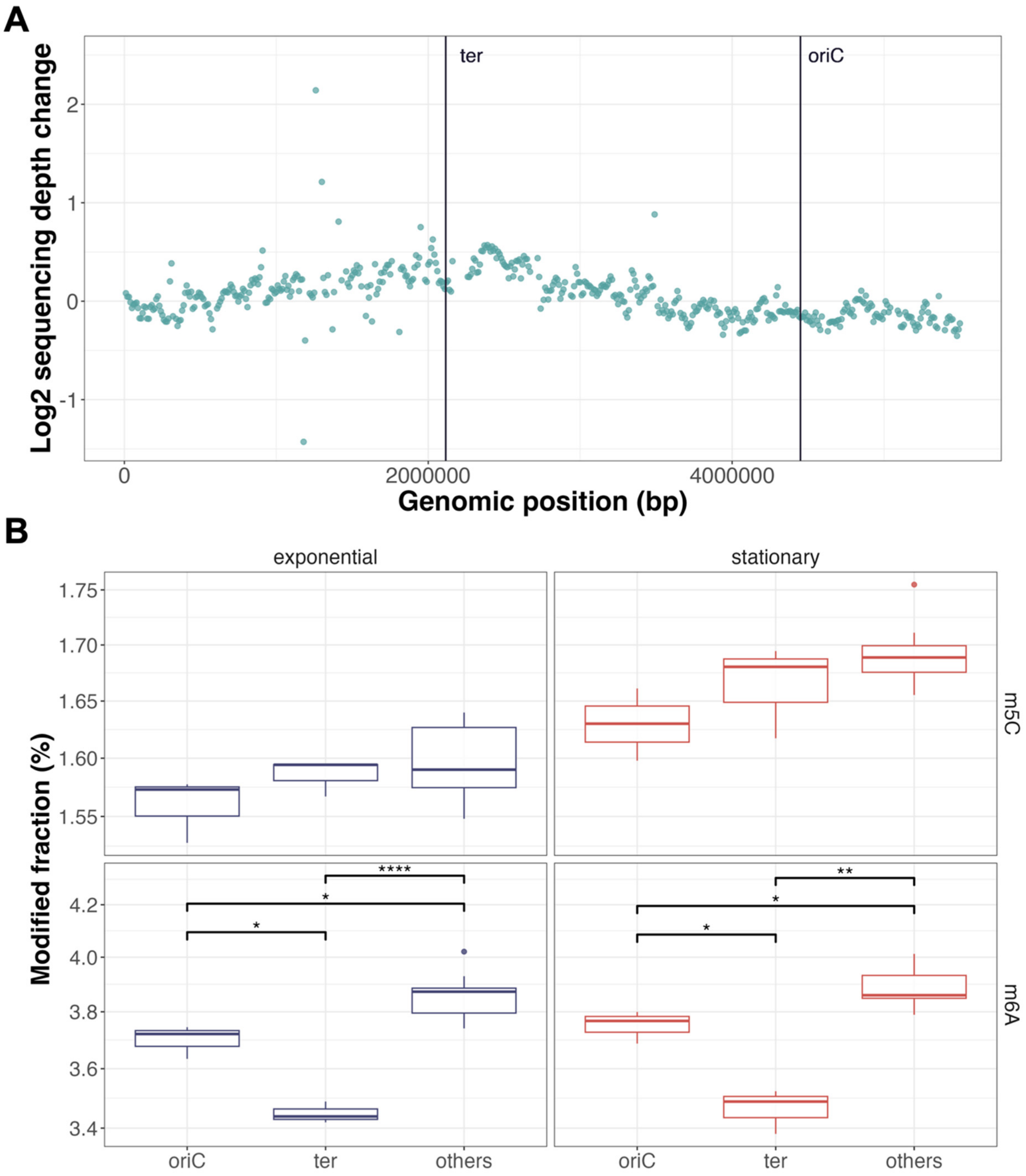

Figure S18. DNA sequencing depth and DNA methylation analyses of the *Escherichia coli* validation dataset. (A) Sequencing depth changes of each 10-kb window across the genome between the exponential and stationary phases; (B) DNA methylation levels within the DNA replication origin and terminus 100-kb flanking regions, as well as the regions outside these windows. Analysis here only included replicates 1, 2, and 3 with DNA and RNA sequencing data in *E. coli*. The RefSeq genome (GCF_000008865) was used for

the analysis. Only the data from the chromosome NC_002695.2 were included in graph B. The log2 value change is the base-2 logarithm of the stationary value relative to the exponential value. A unpaired t-test was used to compare the DNA methylation level between regions. *ter*: DNA replication terminus or its 100-kb flanking region. *oriC*: DNA replication origin or its 100-kb flanking region. Others: regions outside the DNA replication origin and terminus 100-kb flanking regions.. *P-values* < 0.05: *; *P-values* < 0.01: **; *P-values* < 0.001: ***.

Figure S19

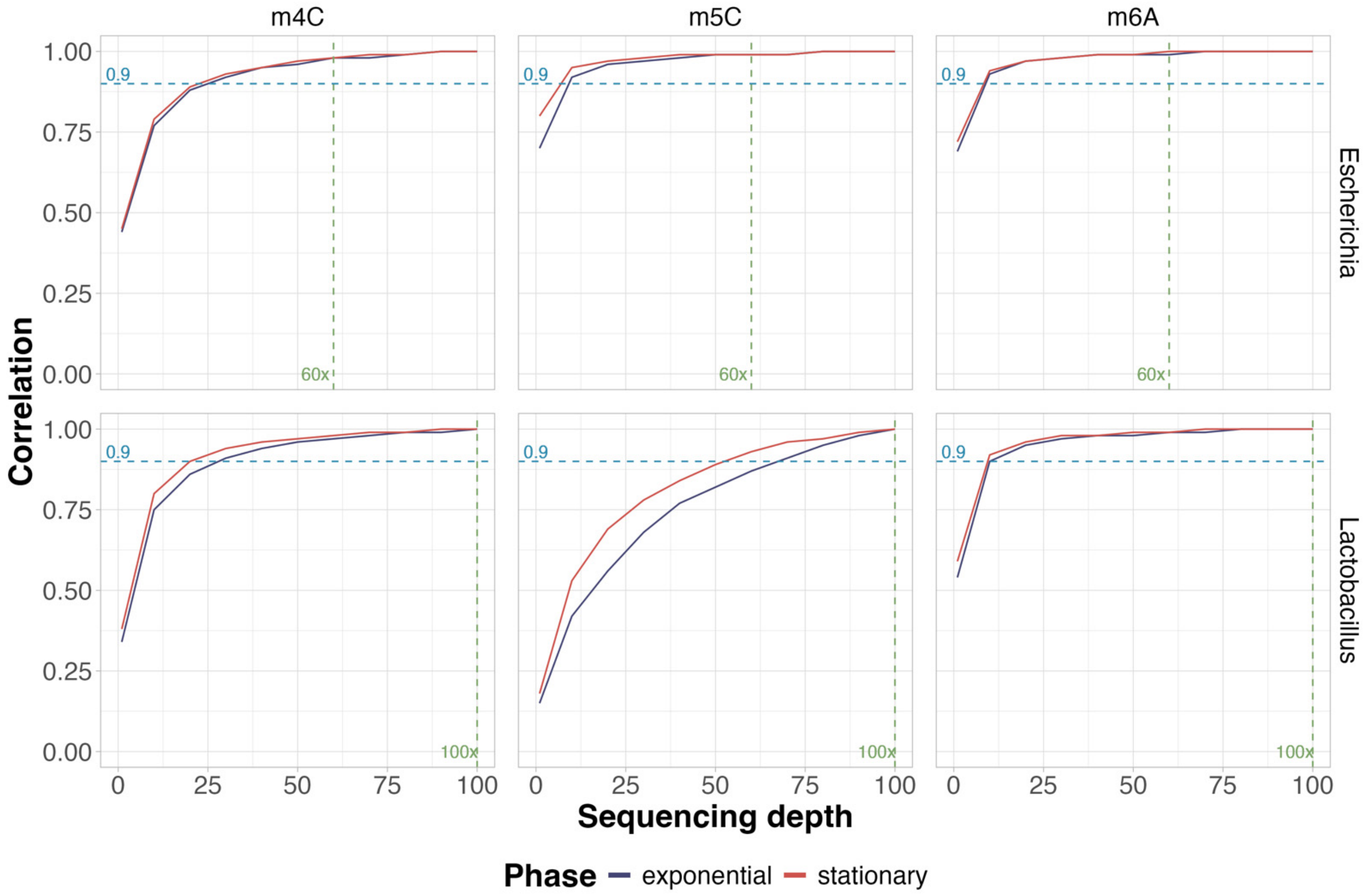

Figure S19. Base-level DNA methylation correlations of different sequencing depths with 100x coverage across three DNA methylation. The assembled genomes were used for the analysis. Only samples with over 100x coverage in the dataset were used for this analysis. Samples with 100x coverage were used as a benchmark. Genomic loci present in all subsampled datasets were used without specifying motifs. The Pearson correlation coefficient was calculated to evaluate the correlation between different sequencing depths with 100x coverage using the base-level DNA methylation proportions. m6A: N6-methyladenine. m4C: N4-methylcytosine. m5C: 5-methylcytosine.

Figure S20

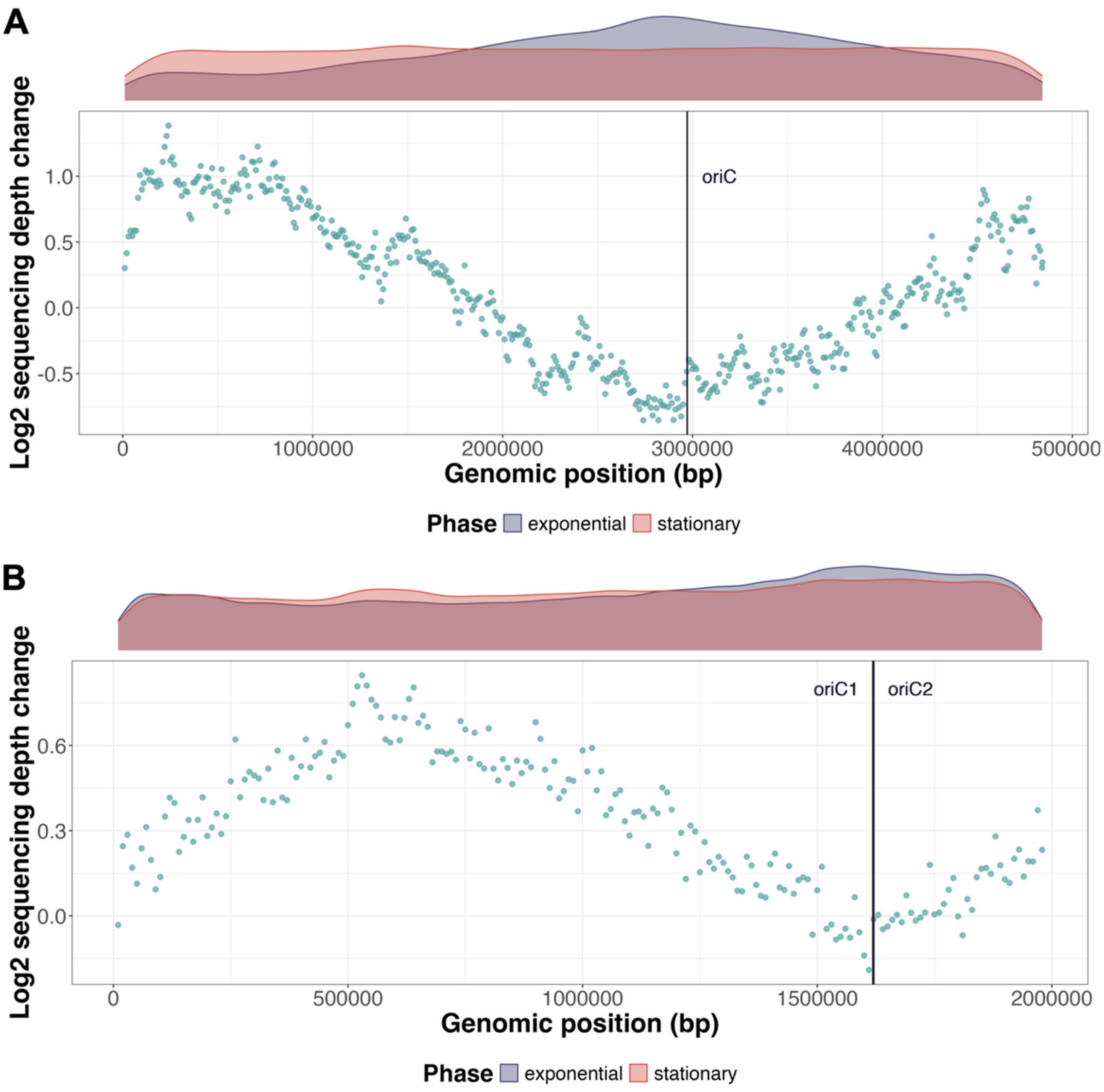

Figure S20. Sequencing depth changes of each 10-kb window across the genome between the exponential and stationary phases in (A) *Escherichia coli* and (B) *Lactobacillus acidophilus*. The assembled genomes were used for the analysis. The side plot on the top of each graph is the DNA sequence start site density across the assembled genome. Only the data from contig 1 were included. The log2 value change is the base-2 logarithm of the stationary value relative to the exponential value. The Spearman correlation coefficient was calculated to estimate the correlation between average DNA length and read number. A paired t-test was used to compare the average length and read number between different phases. *ter*: DNA replication terminus. *oriC*: DNA replication origin. *P-values* < 0.05: *; *P-values* < 0.01: **; *P-values* < 0.001: ***.

Figure S21

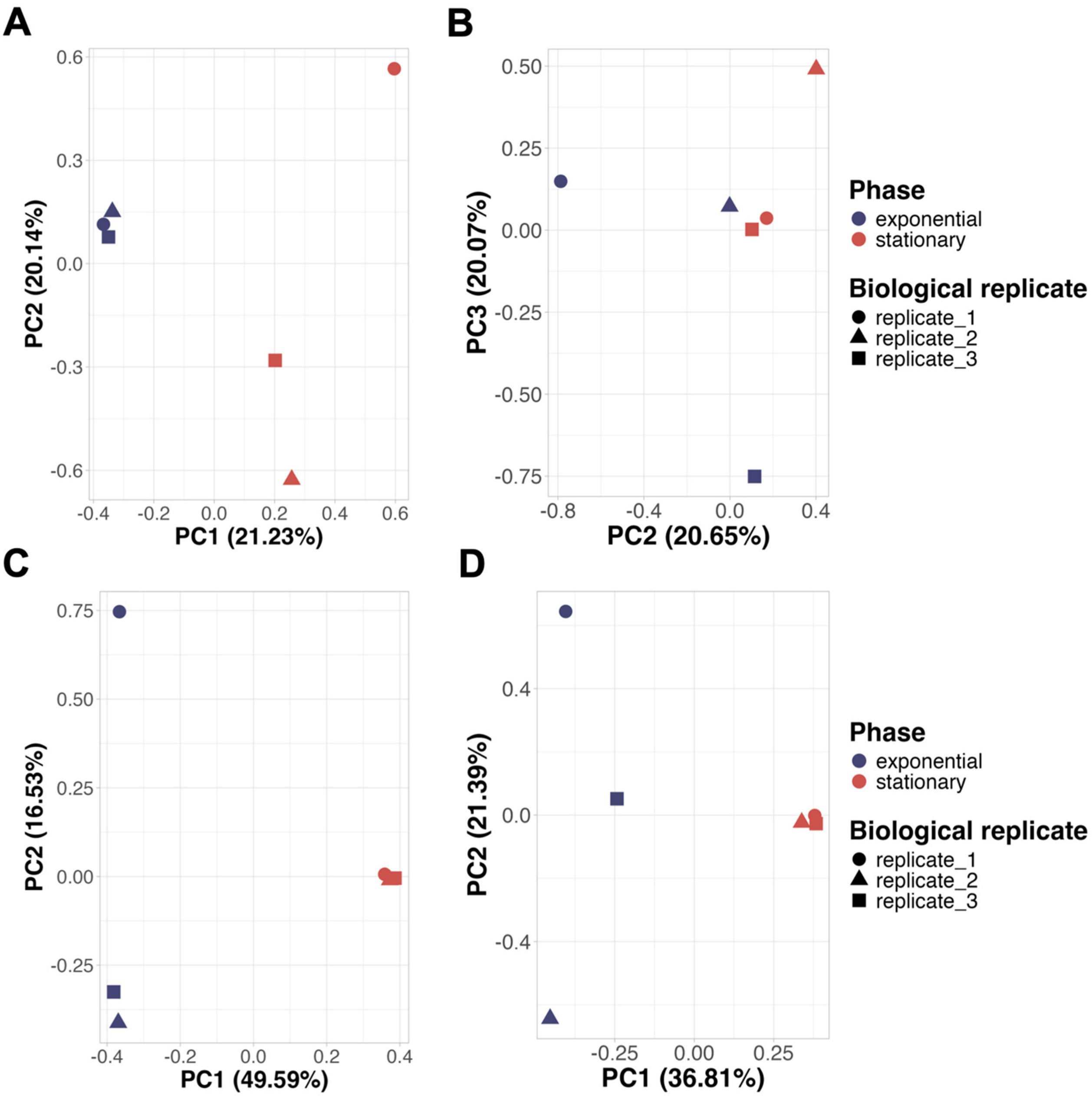

Figure S21. Principal component analysis on DNA methylation proportions in *Escherichia coli* (A, C) and *Lactobacillus acidophilus* (B, D). Panels A and B show results from locus-based methods, while panels C and D represent motif-based methods. The assembled genomes were used for the analysis.

Figure S22

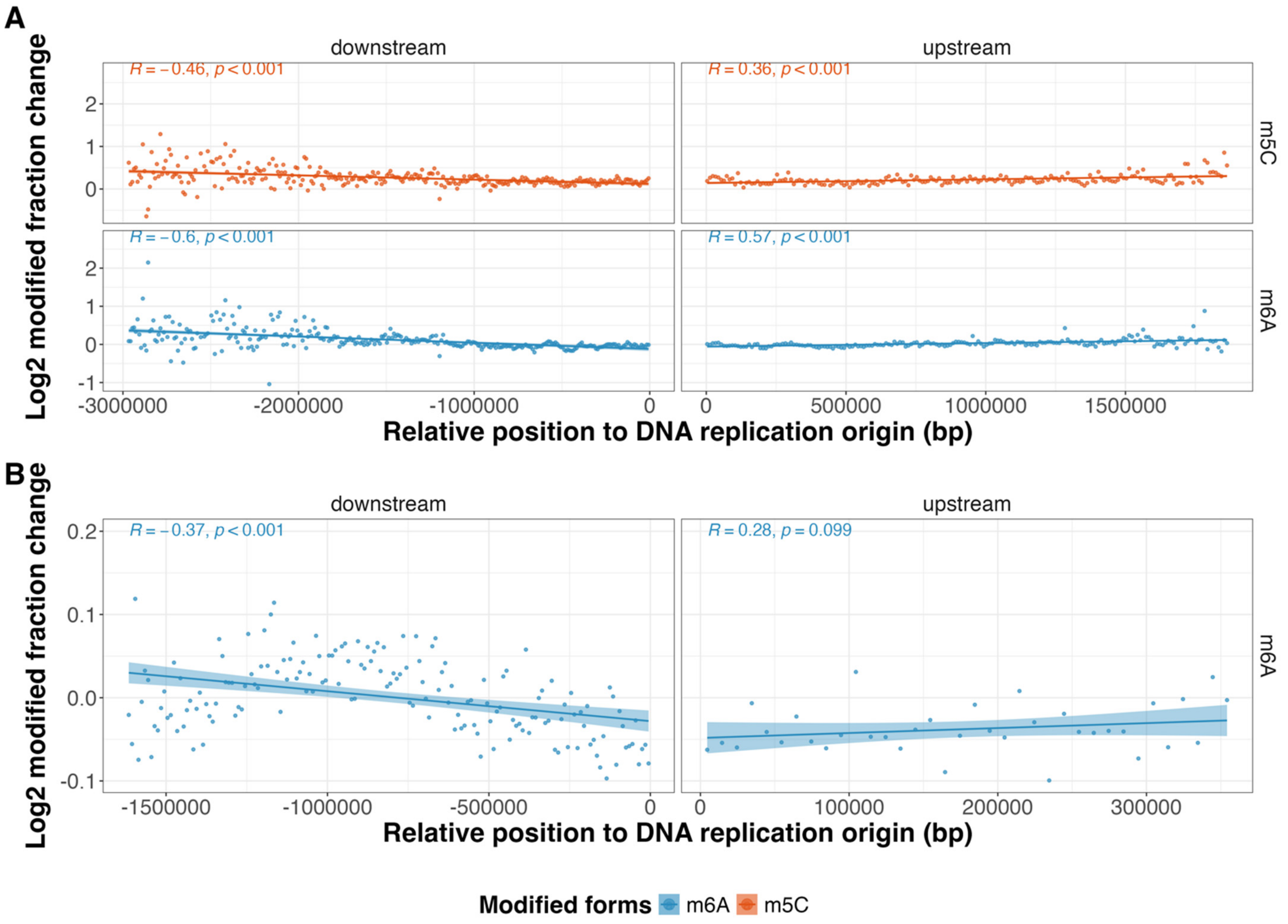

Figure S22. DNA methylation level changes across the genome of (A) *Escherichia coli* and (B) *Lactobacillus acidophilus* using the locus-based approach. DNA methylation level changes of each 10-kb window between the exponential and stationary phases around the DNA replication origin. The assembled genomes were used for the analysis. Only the data from contig 1 were included. The log2 modified fraction change is the base-2 logarithm of the stationary value relative to the exponential value. The Spearman correlation coefficient was calculated to estimate the relation between distance to the DNA replication terminus and log2 modified fraction change. m6A: N6-methyladenine. m5C: 5-methylcytosine.

Figure S23

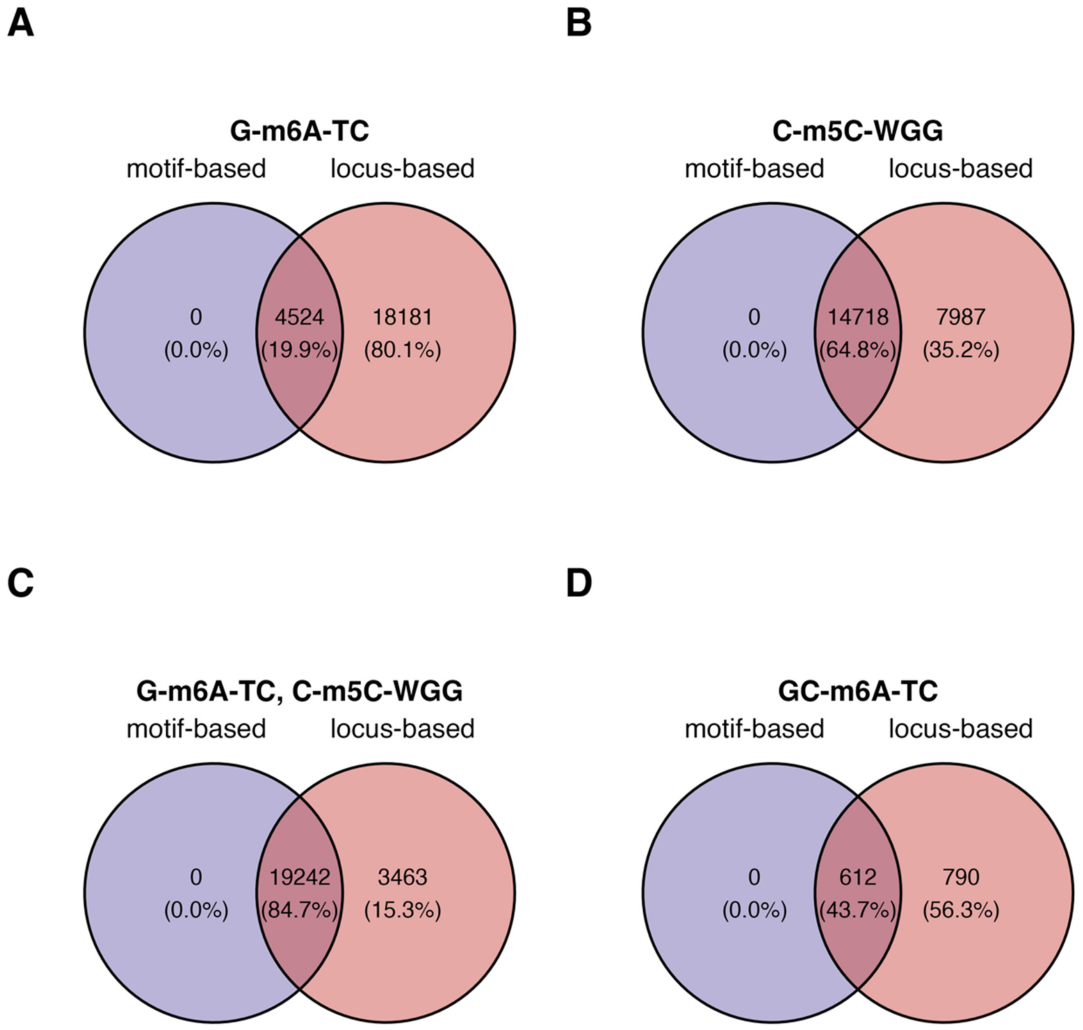

Figure S23. The shared differentially methylated positions in *Escherichia coli* (A, B, and C) and *Lactobacillus acidophilus* (D) between motif-based and locus-based approaches. The assembled genomes were used for the analysis. The title in each graph indicated the motifs included in the motif-based approach. A Fisher's Exact Test was performed to evaluate the identified position overlap significance between the motif-based and locus-based approaches. m6A: N6-methyladenine. m5C: 5-methylcytosine.

Figure S24

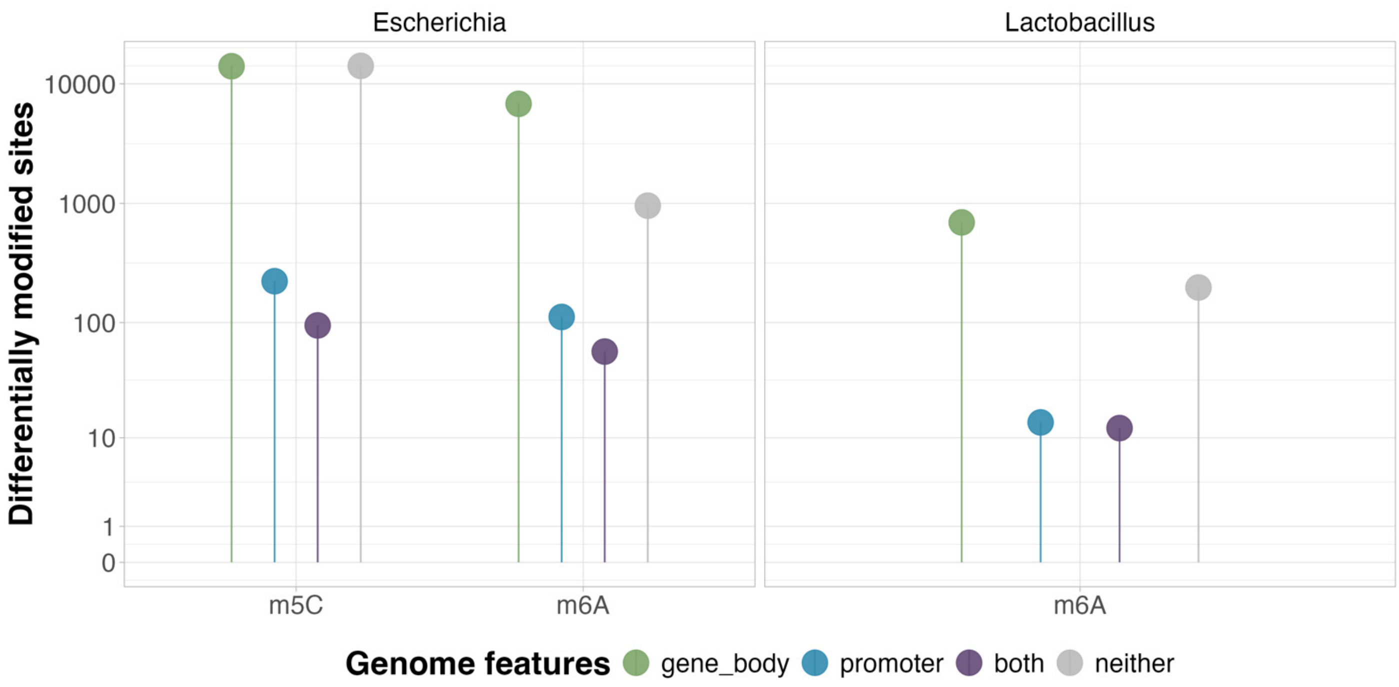

Figure S24. Differentially methylated position distribution in *Escherichia coli* and *Lactobacillus acidophilus* using the locus-based approach. The assembled genomes were used for the analysis. The promoter region was defined as the -35 to -7 frame upstream of the open reading frame start site, while the gene body referred to the region from the open reading frame start to the end site. The differentially methylated sites were normalized by the background GC content. m6A: N6-methyladenine. m5C: 5-methylcytosine.

Figure S25

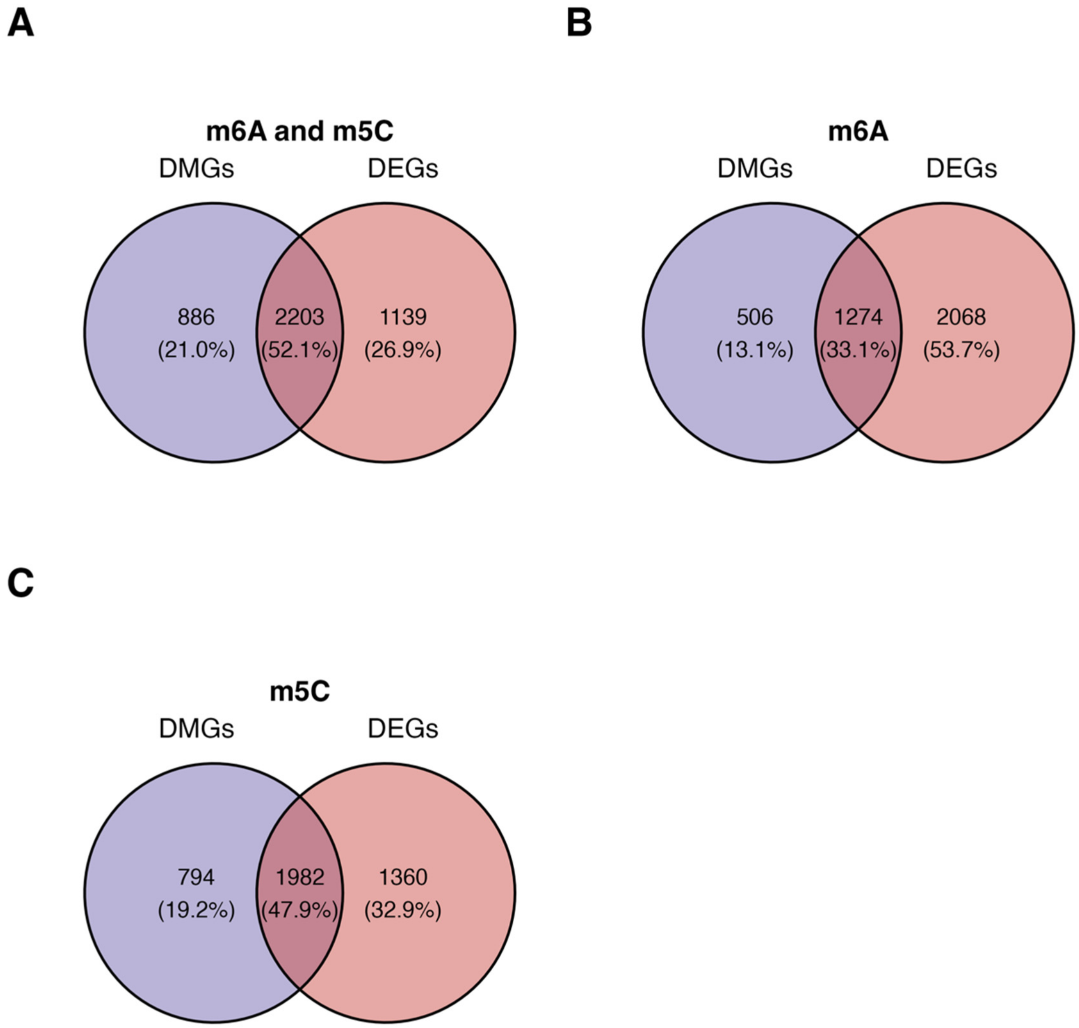

Figure S25. The shared gene number between the locus-based DNA methylation and transcriptomic profiles of *Escherichia coli* in (A) N6-methyladenine and 5-methylcytosine, (B) N6-methyladenine, and (C) 5-methylcytosine. Analysis here only included replicates 1, 2, and 3 with DNA and RNA sequencing data in *E. coli*. The assembled genome was used for the analysis. A Fisher's Exact Test was performed to evaluate the gene overlap significance between the DNA and RNA datasets. m6A: N6-methyladenine. m5C: 5-methylcytosine.

Figure S26

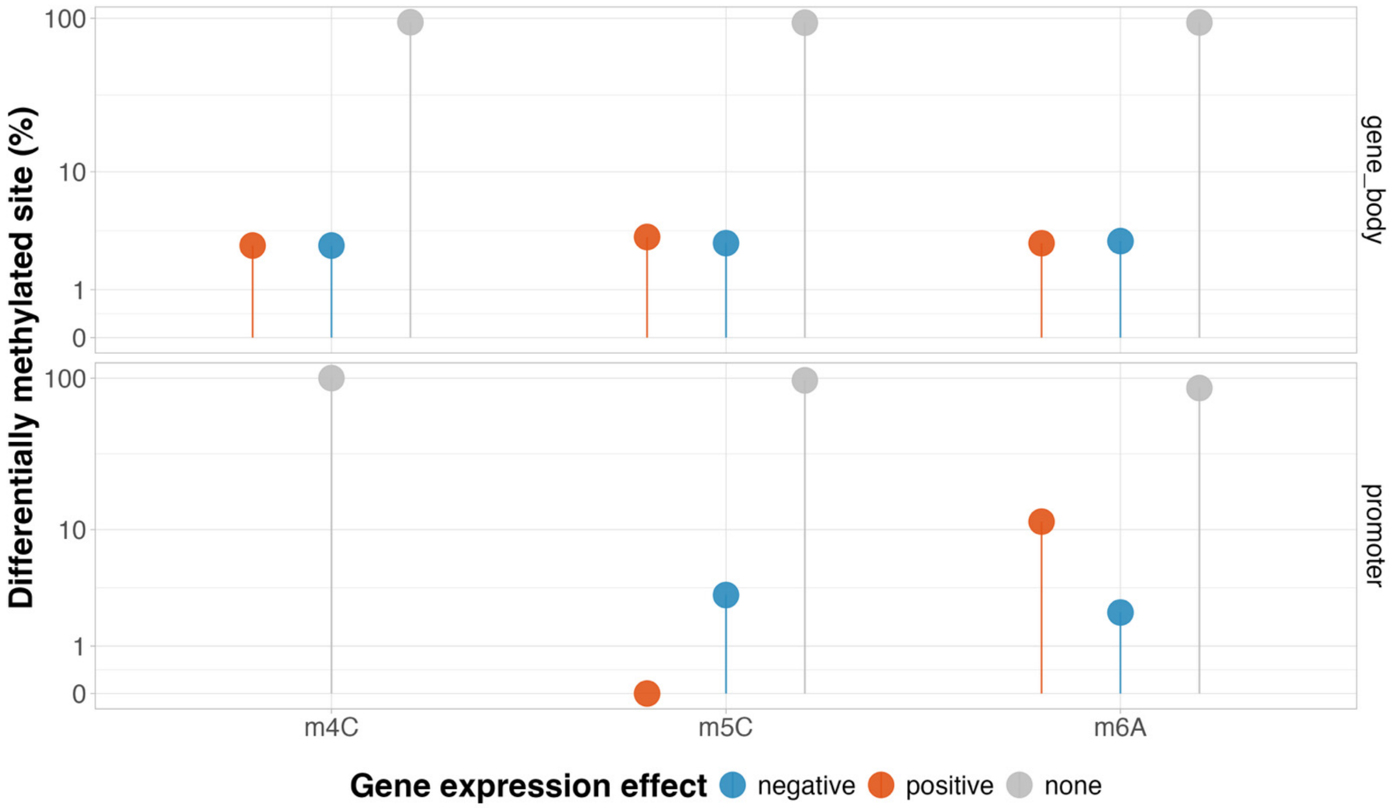

Figure S26. The proportions of differentially methylated genomic sites by gene expression effects in *Escherichia coli* including three DNA methylation forms. Analysis here only included replicates 1, 2, and 3 with DNA and RNA sequencing data in *E. coli*. The RefSeq genome (GCF_000008865) was used for the analysis. Results were derived from the locus-based approach. The coverage threshold for the DNA methylation matrix was 0x. The promoter region was defined as the -35 to -7 frame upstream of the open reading frame start site, while the gene body referred to the region from the open reading frame start to the end site. m6A: N6-methyladenine. m5C: 5-methylcytosine. m4C: N4-methylcytosine.
